# Novel Phosphatase SSU72 Drives Malignant Progression in Colorectal Cancer via PERK*^Thr982^* Dephosphorylation Mediated Endoplasmic Reticulum Stress Inactivation

**DOI:** 10.7150/ijbs.129555

**Published:** 2026-07-22

**Authors:** Dongming Liu, Yuchao He, Wenshuai Chen, Zhonghao Sun, Wenchen Gong, Yu Wang, Jiaqi Ni, Yi Luo, Liwei Chen, Changfu Liu, Hua Li, Hua Guo

**Affiliations:** 1 Tianjin Medical University Cancer Institute & Hospital, National Clinical Research Center for Cancer, Tianjin's Clinical Research Center for Cancer, State Key Laboratory of Druggability Evaluation and Systematic Translational Medicine, Tianjin Key Laboratory of Digestive Cancer, Tianjin, 300060, China.; 2 Department of Hepatobiliary Cancer, Liver Cancer Research Center, Tianjin Medical University Cancer Institute and Hospital, Tianjin, 300060, China.; 3 Department of Tumor Cell Biology, Tianjin Medical University Cancer Institute and Hospital, Tianjin, 300060, China.; 4 Geneplus-Beijing Institute, Beijing, 102206, China.; 5 Department of Thoracic Oncology, Lung Cancer Diagnosis and Treatment Center, Tianjin Medical University Cancer Institute and Hospital, 300060, Tianjin, China.; 6 Department of Pathology, Tianjin Medical University Cancer Institute and Hospital, Tianjin, 300060, China.; 7 Department of Interventional Therapy, Tianjin Medical University Cancer Institute and Hospital, Tianjin, 300060, China.; 8 Department of Endoscopy, Tianjin Medical University Cancer Institute and Hospital, Tianjin, 300060, China.

**Keywords:** colorectal cancer, SSU72, endoplasmic reticulum stress, PERK, dephosphorylate

## Abstract

Colorectal cancer, the third most prevalent malignancy worldwide, presents significant clinical challenges due to late-stage diagnosis, therapeutic resistance, and molecular heterogeneity. Here, we identify the phosphatase SSU72 as a catalyst of colorectal cancer progression and elucidate its mechanism. High-throughput sequencing and bioinformatics analysis revealed that SSU72 overexpression was correlated with poor prognosis and found to suppress autophagy and apoptosis. Mechanistically, proteomic, RNA sequencing and single-cell RNA analyses revealed SSU72's interaction with PERK, a key endoplasmic reticulum stress regulator. Co-immunoprecipitation, immunofluorescence, and rescue experiments demonstrated that SSU72 dephosphorylated PERK at *Thr982*, inactivating endoplasmic reticulum stress signaling. *In vivo* experimental validation confirmed that SSU72-mediated suppression of endoplasmic reticulum stress promoted tumor aggressiveness. Clinically, SSU72 serves as a novel biomarker for colorectal cancer poor prognosis, and targeting its phosphatase activity to restore endoplasmic reticulum stress represents a therapeutic strategy for SSU72-high tumors. This study uncovers SSU72's dual role: beyond its canonical functions in transcription and cell cycle regulation, it directly modulates endoplasmic reticulum stress to promote colorectal cancer malignancy. Our findings address critical deficiencies in understanding colorectal cancer heterogeneity and propose SSU72 as a prognostic biomarker and viable target for precision therapy.

## Introduction

Colorectal cancer (CRC) is the third most common cancer globally and the second leading cause of cancer-related mortality^1^. The clinical management of CRC faces significant challenges. Early symptoms are often insidious, and approximately 20%-30% of cases are diagnosed at stage IV with metastatic disease^2^, ^3^. Advanced tumors involving adjacent organs or distant metastases pose substantial difficulties in achieving curative surgical resection^4^. Standard chemotherapeutic regimens are susceptible to developing drug resistance^5^. Additionally, given the highly heterogeneous nature of CRC, molecular subtyping is critical for precision therapy. However, effective targeted therapies remain unavailable for certain subtypes, highlighting the urgent need to identify novel molecular biomarkers^3^.

Through high-throughput sequencing and large-scale bioinformatics database screening, we identified the phosphatase SSU72 as a key factor strongly associated with poor prognosis in CRC. In terms of tumor biological behavior, SSU72 overexpression predominantly suppresses tumor autophagy and apoptosis. SSU72 was initially characterized as a phosphatase for the carboxy-terminal domain of RNA polymerase II, responsible for removing phosphorrylation at Ser5/Ser7 residues, thereby regulating transcription termination, RNA splicing, and 3'-end processing^6^. SSU72 also plays a role in cell cycle regulation by dephosphorylating cell cycle-associated proteins, modulating DNA replication initiation, and maintaining genomic stability^7^. Under DNA damage or replication stress, SSU72 may participate in the activation of checkpoint signaling^8^. Notably, our literature review revealed that current research on SSU72 predominantly focuses on its phosphatase-dependent roles in RNA metabolism, transcriptional regulation, and cell cycle control, with only two published studies exploring its association with malignant tumors. Therefore, we conducted further investigations integrating the intrinsic phosphatase function of SSU72 with its effect on the malignant progression of CRC.

To elucidate the molecular mechanism by which SSU72 influences the malignant progression of CRC, we combined proteomic sequencing and single-cell database validation to identify the endoplasmic reticulum (ER) stress pathway as a critical signaling axis. ER stress plays a pivotal role in the malignant progression of tumors, and the identification of therapeutic targets based on the ER stress mechanism is crucial for addressing poor cancer prognosis. Through rescue experiments, immunofluorescence co-localization, co-immunoprecipitation (Co-IP), Western blot, and RT-qPCR analyses, we confirmed PERK as the direct interacting protein of SSU72. Furthermore, by focusing on the phosphatase activity of SSU72, we identified *Thr982* as the specific phosphorylation site on PERK targeted by SSU72-mediated dephosphorylation. These findings collectively establish a functional biological axis termed 'SSU72-PERK*^Thr982^*-ER stress-autophagy/apoptosis'. In final *in vivo* validation experiments, we observed that the SSU72 overexpression-mediated inactivation of ER stress likely serves as a critical signaling pathway promoting malignant progression and metastatic potential in CRC. These findings suggest that SSU72 may serve as a novel molecular biomarker for CRC molecular subtyping. Targeting SSU72 to reverse ER stress inactivation may improve prognosis in the subset of patients with CRC exhibiting high SSU72 expression.

## Materials and Methods

### Patients, tissue specimens, and immunohistochemistry (IHC) staining

Six pairs of fresh-frozen CRC tissues and matched adjacent non-tumor tissues were prospectively collected from Tianjin Medical University Cancer Institute and Hospital from January 2015 to December 2020. For SSU72 expression analysis, tissue microarrays (HColA180Su22, Shanghai Qutdo Biotech, Shanghai, China) containing 94 primary CRC tissues and 86 matched normal tissues were used to analyze levels of SSU72. IHC staining was performed as previously described^9^. All specimens were histologically confirmed as CRC by two board-certified pathologists using hematoxylin and eosin-stained sections, with tumor staging performed according to the 8th edition AJCC guidelines. Patients with preoperative neoadjuvant therapy (radiotherapy/chemotherapy) were excluded from this study.

### Cell culture and cell transfection

HCT-8 and HCT-116 cell lines (American Type Culture Collection, ATCC) were cultured in RPMI-1640 medium (NY, USA, Corning) containing 10% FBS (PAN-Seratech, Edenbach, Germany) and 1% PS (HyClone, Logan, UT, USA) at 37 °C under 5% CO₂. Packaging plasmids (VSVG and ΔR) and expression plasmids (sh-Ctrl and sh-SSU72) were transfected into HEK293T cells using Lipofectamine 2000 (Invitrogen, CA, USA) to obtain lentiviral particles. The HEK293T cells were used to produce the lentiviruses. HCT-8 and HCT-116 cells were prepared for transfection. Polybrene (Solarbio, Beijing, China) was used as the transfection reagent. A stably transfected cell line was obtained under puromycin (Gibco, CA, USA) and hygromycin B (Roche, Basel, Switzerland) selection.

### Statistical analyses

All statistical analyses were performed using GraphPad Prism (version 9.5.1) and R software (version 4.4.3). Unless otherwise specified, data are presented as mean ± standard deviation, and all *in vitro* experiments were independently repeated at least three times. Comparisons between two groups were performed using paired or unpaired Student's 't'-test. Comparisons among multiple groups were performed using one-way analysis of variance (ANOVA) followed by Bonferroni's post hoc test. Correlation analyses were performed using Spearman's rank correlation test. Survival curves were generated using the Kaplan-Meier method, and differences in survival between groups were assessed using the log-rank test. Univariate and multivariate Cox proportional hazards regression models were used to evaluate the independent prognostic impact of relevant factors. All statistical tests were two-sided. The significance levels for *p* values were defined as follows: *p < 0.05, **p < 0.01, ***p < 0.001, ****p < 0.0001, ns indicates no statistically significant difference.

The details about other assays were described in the Supplementary Materials and Methods.

## Results

### Novel phosphatase SSU72 promotes poor prognosis in CRC

**Figure 1:**
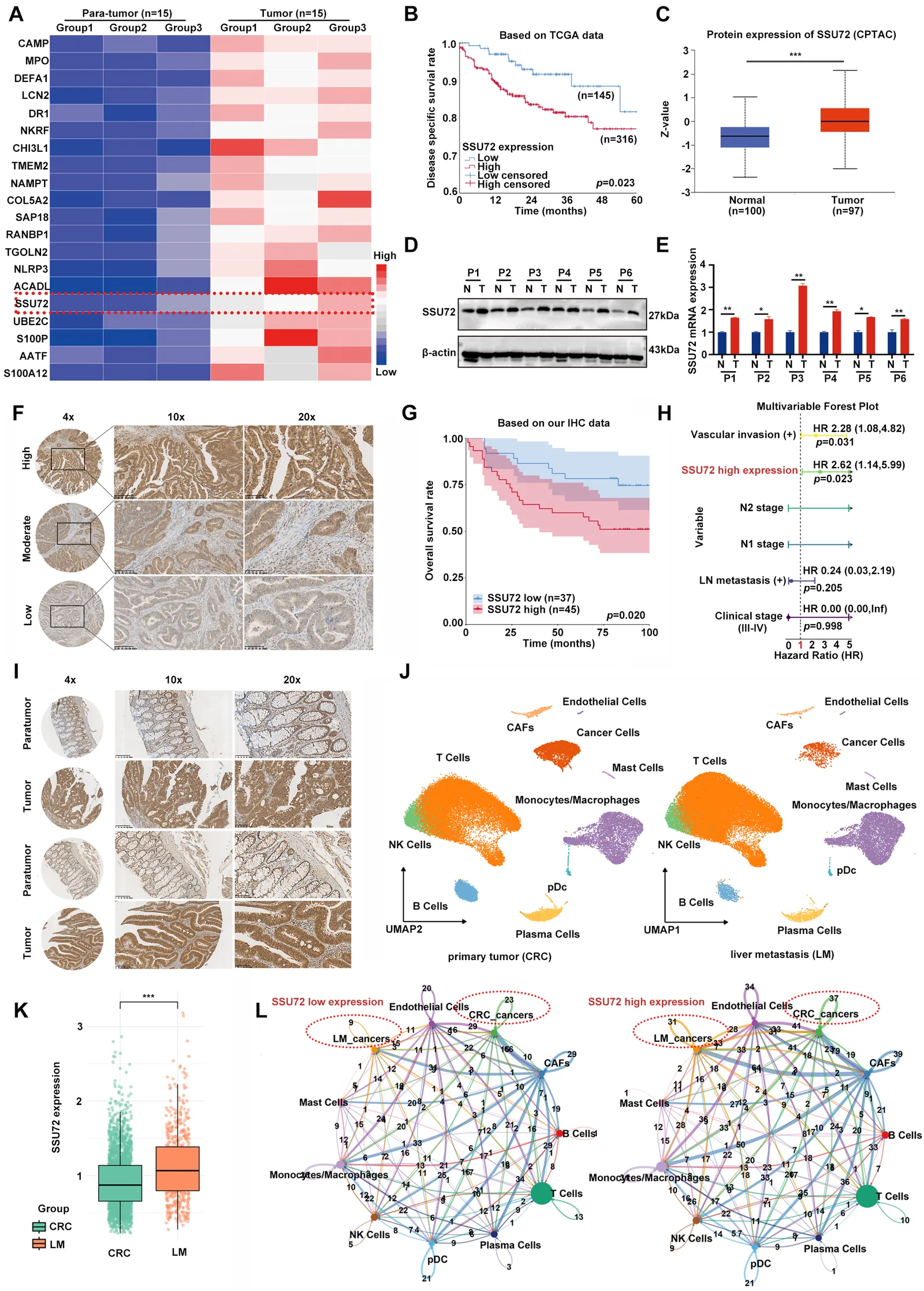
** Novel phosphatase SSU72 promotes poor prognosis in CRC.** A. Differentially expressed proteins in CRC by iTRAQ sequencing. B. Relationship between SSU72 expression and CRC prognosis analyzed using the TCGA database. C. SSU72 expression in CRC tissues detected via the CPTAC proteomic database. D&E. SSU72 expression in paired human CRC tissues measured by RT-qPCR and Western blot. F. Representative IHC staining images showing high, medium, and low SSU72 expression in CRC. G. Univariate survival curve analysis of OS in CRC based on SSU72 expression. H. Multivariate analysis of clinical factors influencing CRC prognosis. I. Representative IHC staining of SSU72 in CRC and adjacent non-tumor tissues. J. Cell clustering in CRC and LM based on the GSE178318 single-cell dataset. K. Differential expression of SSU72 in CRC and LM according to the GSE178318 dataset. L. Cell-cell interaction networks across different SSU72 expression levels.

We selected 15 patients with CRC and similar clinical baseline characteristics. The cancer tissues and adjacent non-cancerous tissues from every five patients were grouped into one cohort, resulting in three cancer tissue cohorts and three para-tumor tissue cohorts for iTRAQ-based proteomic sequencing (detailed information was shown in Supplementary Table 2). Proteins meeting the screening criteria of a fold change (FC) >2 and a *p* value <0.05 were identified as differentially expressed proteins, with the top 20 ranked overexpression proteins (the smaller the *p* value, the higher the rank) shown in Figure 1A. Further analysis using the Kaplan-Meier Plotter online database (*https://www.kmplot.com/analysis/*) screened for proteins associated with poor prognosis in CRC (Supplementary Figure 1A-T). Only CAMP (Supplementary Figure 1A) and SSU72 (Supplementary Figure 1P) were found to promote adverse clinical outcomes. Finally, validation via the UALCAN database (*https://ualcan.path.uab.edu/*) confirmed that SSU72 was overexpressed in cancer tissues and significantly affected poor prognosis (Supplementary Figure 1U), whereas CAMP (Supplementary Figure 1V) showed no differential expression.

We further validated our findings using the TCGA database with a large sample size, which similarly revealed a significant association between *SSU72* and poor prognosis in CRC within a large cohort of 461 cases (Figure 1B). Subsequent analyses using the CPTAC proteomic database (Figure 1C), the GSE163974 single-cell sequencing database (Supplementary Figure 2A), the TCGA database, and multiple GEO datasets (GSE113513/GSE21825/GSE37182/GSE44076/GSE87211) (Supplementary Figure 2B) confirmed that the phosphatase *SSU72* functions as a novel oncogene. Building upon the sequencing and bioinformatics database results, we confirmed the findings using Western blot (Figure 1D) and RT-qPCR (Figure 1E) on paired cancer and adjacent tissues from six patients with CRC, confirming consistency with the aforementioned data. As shown in Figure 1F, the expression of SSU72 in CRC tissues was detected by immunohistochemistry (IHC). On the basis of staining intensity, tissues could be classified into high, medium, and low expression levels. Tissue microarray analysis demonstrated that high SSU72 expression served as an independent risk factor (HR = 2.62, *p* = 0.023) for poor prognosis in CRC, whereas vascular invasion was an independent risk factor (Figure 1G&H, Supplementary Figure 2C).

We further examined the expression pattern of SSU72 across the spectrum from para-tumor tissues to cancerous tissues, including primary and metastatic tumors. IHC staining revealed markedly lower SSU72 expression in para-tumor tissues compared with matched cancerous areas (Figure 1I). In further analysis of the GSE178318 single-cell dataset, we reclustered the tumor cells from epithelial cells based on copy number variation, and then categorized cells into primary (CRC) and liver metastasis (LM) tumor cells based on their sample origin (Supplementary Figure 2D&E, Figure 1J). The results revealed elevated SSU72 expression in LM cells (Figure 1K). Compared with the SSU72-low group, the SSU72-high group exhibited stronger cell-cell interactions between CRC and LM cancer cells, suggesting that high SSU72 expression may represent a key molecular condition driving CRC progression (Figure 1L). Using the GSE132465 dataset for validation, we performed copy number variation inference based on the authors' original annotation. Tumor cells were identified as epithelial cells with high copy number scores (Supplementary Figure 2F-H). SSU72 expression in tumor cells was then examined across different tumor stages. The results showed that SSU72 expression was most strongly associated with the T stage, which reflects tumor progression in the TNM classification. Moreover, SSU72 expression levels increased progressively with higher T stages (Supplementary Figure 2I). These results demonstrated that* SSU72* acts as a novel oncogene contributing to adverse prognosis in CRC.

### SSU72 promotes malignant transformation in CRC by suppressing tumor cell apoptosis and autophagy

**Figure 2:**
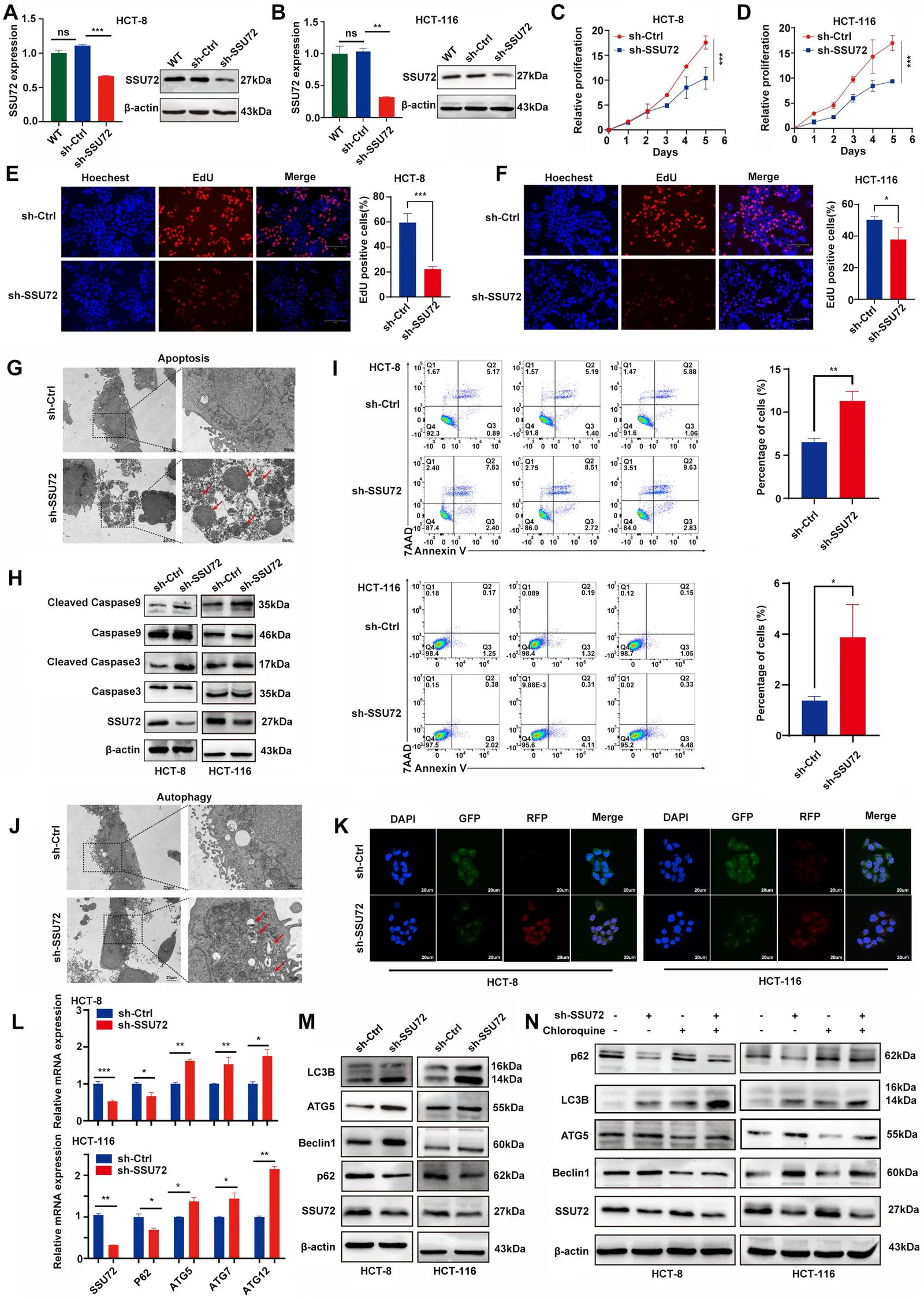
** SSU72 promotes malignant transformation in CRC by suppressing tumor cell apoptosis and autophagy.** A&B. Establishment and validation of stable SSU72 knockdown and control cell lines (HCT-116 and HCT-8) via Western blot and RT-qPCR. C-F. CCK-8 and EdU proliferation assay evaluating the relationship between SSU72 expression levels and cell proliferation capacity. G. Detection of apoptotic bodies across different SSU72 expression levels by TEM. H. Changes in apoptosis markers after SSU72 knockdown measured by Western blot. I. Apoptosis capability following SSU72 knockdown assessed by flow cytometry apoptosis assay. J. Observation of autophagosomes across different SSU72 expression levels using TEM. K. Differences in autophagic flux among CRC cells with varying SSU72 expression levels. L&M. Changes in autophagy markers after SSU72 knockdown detected by RT-qPCR and Western blot. N. Restoration of autophagy marker levels after chloroquine treatment analyzed by Western blot.

To further investigate the molecular mechanisms by which SSU72 promotes malignant progression in CRC, we established stable sh-SSU72 and control cell lines in HCT-116 and HCT-8 cells, confirmed by RT-qPCR and Western blot (Figure 2A&B).

These models provided a solid foundation for subsequent functional studies. Analysis of two single-cell sequencing datasets (GSM5688706 and GSM5688709) revealed a negative correlation between SSU72 expression and tumor cell apoptosis (Supplementary Figure 3A&B, D&E). In HCT-116 and HCT-8 cell lines, CCK-8 and EdU assays showed that SSU72 knockdown significantly reduced tumor cell viability (Figure 2C-F). TEM observation further demonstrated increased apoptotic body formation in sh-SSU72 cells (Figure 2G). Western blot analysis indicated upregulated levels of cleaved caspase-3 and cleaved caspase-9 in sh-SSU72 cells, whereas total caspase-3 and caspase-9 protein levels remained unchanged (Figure 2H). Flow cytometry-based apoptosis assays confirmed a marked increase in apoptotic CRC cells following SSU72 suppression (Figure 2I). Collectively, these findings demonstrated that SSU72 promotes malignant progression in CRC by inhibiting tumor cell apoptosis.

During TEM observation of apoptotic bodies, we unexpectedly discovered an increased number of autophagosomes in sh-SSU72 cells (Figure 2J). This result suggested that SSU72 may influence CRC malignant progression through mechanisms beyond apoptosis alone, prompting further investigation into its role in tumor cell autophagy. First, correlation analysis using the aforementioned single-cell datasets revealed a negative association between SSU72 expression levels and tumor cell autophagy (Supplementary Figure 3C&F). Second, we transfected HCT8 and HCT116 cell lines with an mRFP-GFP-LC3 dual-fluorescence reporter system. Fluorescence microscopy showed that sh-SSU72 cells exhibited significantly weakened green fluorescence signals and enhanced red fluorescence signals (Figure 2K), indicating that sh-SSU72 promotes autophagosome-lysosome fusion and enhances autophagic flux, thereby activating autophagy in CRC cells.

RT-qPCR and Western blot analyses of classical autophagy markers demonstrated decreased p62 levels and upregulated ATG5/ATG7/ATG12/Beclin1 expression in sh-SSU72 CRC cells (Figure 2L&M). To validate the reversibility of this process, we treated knockdown and control CRC cells with 25 μM chloroquine (CQ), a known autophagy inhibitor that blocks autophagosome-lysosome fusion by suppressing lysosomal acidification^11^. Western blot results revealed that CQ treatment significantly increased LC3B and p62 levels but reduced Beclin1 and ATG5 expression in sh-SSU72 CRC cells. These findings confirmed that SSU72 knockdown promotes autophagosome formation and reduces autophagic substrate (p62) accumulation, thereby inducing autophagy (Figure 2N).

To ensure the scientific rigor and comprehensiveness of this study, we further constructed and validated SSU72-overexpressing cells and corresponding vector control cells, as well as an additional sequence of sh-SSU72# and sh-Ctrl cells, and once again confirmed the regulatory role of SSU72 in autophagy and apoptosis in CRC (Supplementary Figure 4A-C&F-H). Furthermore, colony formation and Transwell assays demonstrated that SSU72 influences the malignant phenotype transformation of CRC by affecting cell proliferation and invasion (Supplementary Figure 4D&E&I&J).

### SSU72 promotes malignant progression in CRC by suppressing ER stress

**Figure 3:**
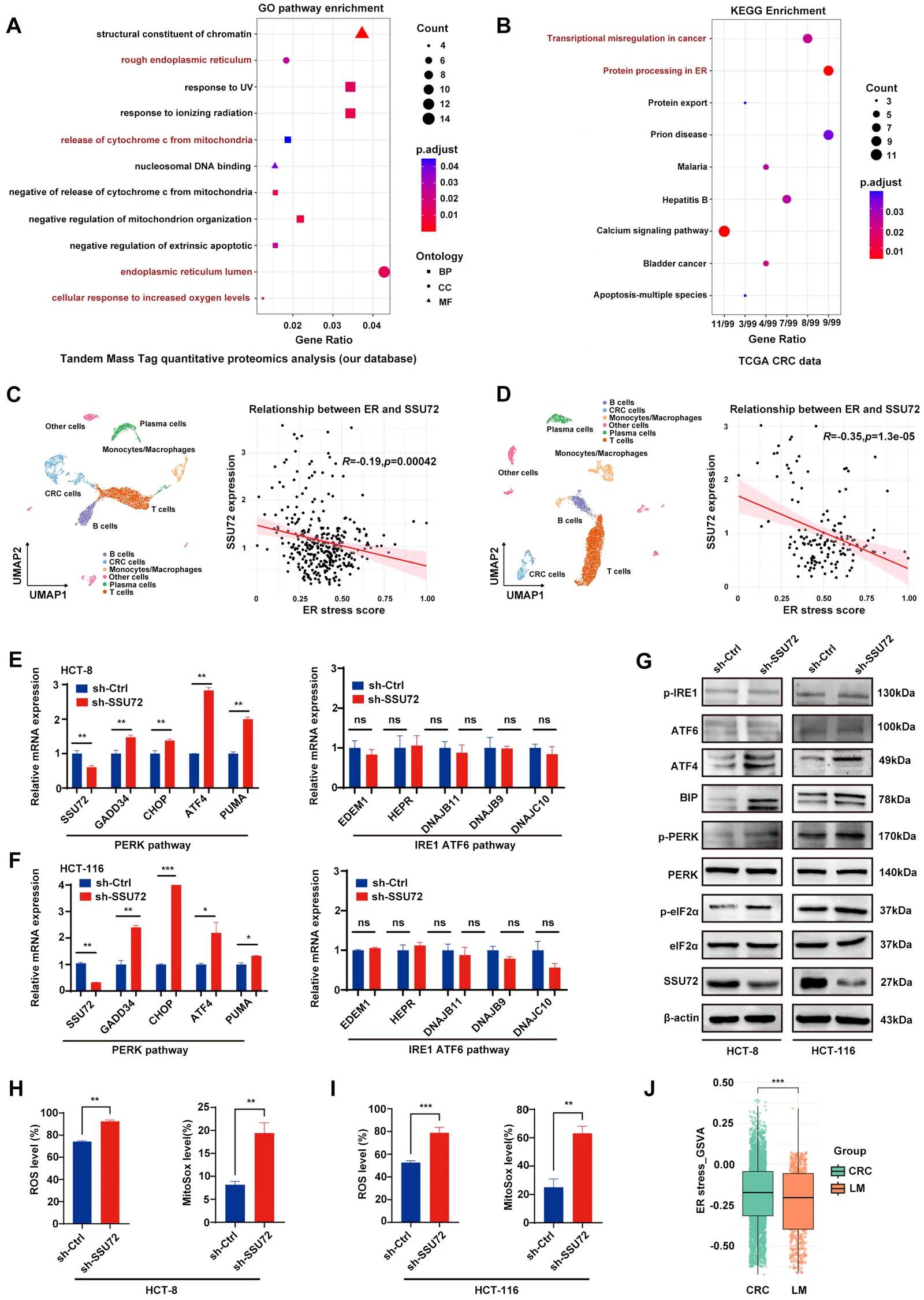
** SSU72 promotes malignant progression in CRC by suppressing endoplasmic reticulum stress.** A. GO analysis of TMT sequencing data identifying signaling pathways closely associated with SSU72 expression levels. B. KEGG analysis based on TCGA data validating the TMT sequencing results. C&D. Relationship between SSU72 expression and ER stress score analyzed using single-cell datasets GSM5688706 and GSM5688709. E&F. Changes in markers of ER signaling pathways (PERK/IRE1/ATF6) after SSU72 knockdown detected by RT-qPCR. G. Western blot validation of the RT-qPCR results. H&I. Alterations in ROS and Mito Sox levels following SSU72 knockdown. J. Differential expression of ER stress score in CRC and LM according to the GSE178318 dataset.

To further elucidate the signaling pathways through which SSU72 regulates apoptosis and autophagy in CRC, we performed tandem mass tag (TMT) based proteomic sequencing on sh-SSU72 and control cell lines. Differential expression analysis, combined with Gene Ontology (GO) functional annotation, revealed that SSU72 knockdown significantly affected cellular components and biological processes related to ER function, including *rough endoplasmic reticulum*, *release of cytochrome c from mitochondria*, *endoplasmic reticulum lumen*, and *cellular response to increased oxygen levels* (Figure 3A). These findings suggested that SSU72 plays a critical role in modulating ER function and associated cellular stress responses.

Building on prior findings, we validated these results using bioinformatics approaches. First, we analyzed CRC datasets from the TCGA database, differential expression analysis was performed based on groups defined by the quartiles of SSU72 expression levels. Differentially expressed genes (DEGs) were then screened using the thresholds of |log_2_ FC| > 0.58 and FDR < 0.05. To specifically investigate the potential involvement of SSU72 in ER related biological processes, these DEGs were further intersected with ER function, which related gene sets obtained from the MSigDB database. Kyoto Encyclopedia of Genes and Genomes (KEGG) pathway enrichment analysis was subsequently performed based on the intersecting genes rather than all DEGs. KEGG pathway enrichment revealed that DEGs were predominantly enriched in pathways such as *protein processing in ER* and *transcriptional misregulation in cancer* (Figure 3B).

Impaired ER function often leads to rapid accumulation of misfolded proteins, triggering ER stress and affecting cell survival and apoptosis^12^. We hypothesized that SSU72 mediates malignant progression in CRC by modulating ER stress levels.

To validate this, we analyzed single-cell datasets (aforementioned GSM5688706 and GSM5688709) and scored CRC cells using an ER stress-related gene set. In this study, the ER stress score was calculated using a curated ER stress related gene set derived from MSigDB. We have now provided the complete gene list in the Supplementary Table 3. Correlation analysis demonstrated a significant negative association between SSU72 expression and ER stress scores (Figure 3C&D), with reduced SSU72 expression correlating with elevated ER stress.

ER stress is a cellular stress response triggered by the accumulation of misfolded proteins, mediated through three signaling pathways: PERK, ATF6, and IRE1 pathways. To identify the specific pathway through which SSU72 knockdown induces ER stress, we first analyzed ER stress-related targeted molecules via RT-qPCR. The results showed upregulated expression of PERK pathway-regulated molecules, including *GADD34*, *CHOP*, *ATF4*, and *PUMA*, whereas targets of the ATF6 and IRE1 pathways (*EDEM1*, *HEPR*, *DNAJB11*, *DNAJB9*, *DNAJC10*) remained unchanged (Figure 3E&F). RT-qPCR detection of *XBP1u* and *XBP1s*—the established standard for IRE1 activation, reflecting the unconventional splicing of *XBP1* mRNA that converts *XBP1u* to *XBP1s*, which revealed no significant changes in either isoform (Supplementary Figure 4K). Similarly, Western blot analysis of ATF6 p90 (full-length) and its cleaved nuclear transcription factor form p50, which represents the standard for ATF6 activation via the processing of ATF6 from p90 to p50, showed no difference between the two protein levels (Supplementary Figure 4L). These results indicate that neither the IRE1 nor the ATF6 branch is markedly activated. Subsequent Western blot analysis confirmed elevated levels of phosphorylated PERK (p-PERK), phosphorylated eIF2α (p-eIF2α), ATF4, and BIP in sh-SSU72 cells, with no alterations in total PERK or eIF2α levels (Fig. 3G). We further analyzed the expression levels of reactive oxygen species (ROS) and mitochondrial ROS (MitoSOX), which are key mediators linking ER stress to mitochondrial dysfunction. SSU72 knockdown significantly upregulated ROS and MitoSOX levels (Figs. 3H and 3I), further corroborating its critical role in modulating ER stress. Comparative analysis of the predefined primary CRC and LM cancer cells in the GSE178318 dataset demonstrated a considerably lower ER stress score in LM cells, indicating a progressive functional impairment of the ER with advancing disease (Fig. 3J). These findings further validated that SSU72 suppresses ER stress in CRC by inhibiting the PERK signaling cascade.

### SSU72 suppresses ER stress in CRC by interacting with PERK and inducing its dephosphorylation

**Figure 4:**
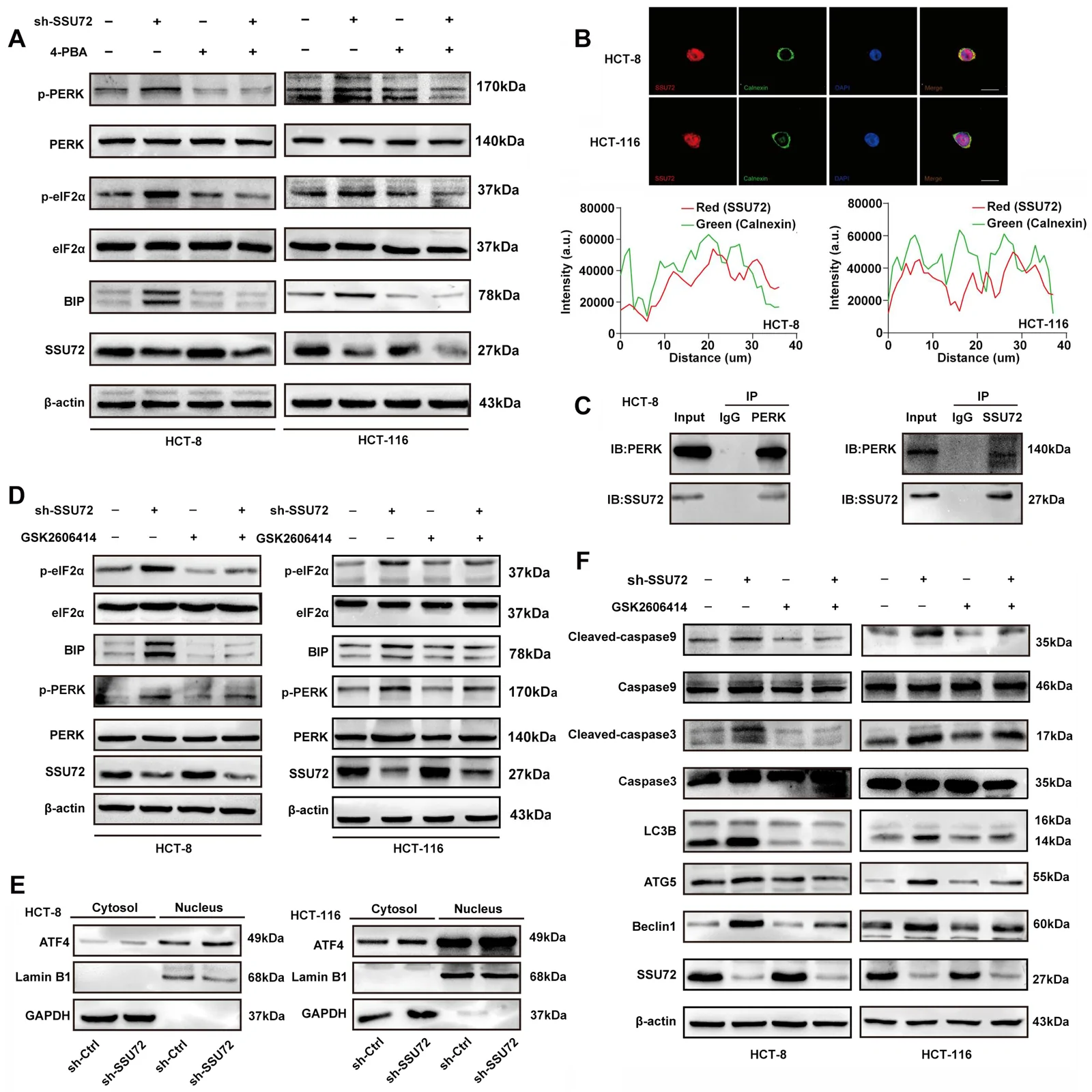
** SSU72 suppresses ER stress in CRC by interacting with PERK and inducing its dephosphorylation**. A. Changes in ER stress levels mediated by SSU72 knockdown were measured following treatment with the ER inhibitor 4-PBA. B. Immunofluorescence assay demonstrating the co-localization relationship between the ER membrane protein Calnexin and SSU72. C. Co-IP confirming the direct interaction between SSU72 and PERK. D. Using the PERK pathway inhibitor GSK2606414, detection of SSU72 knockdown-induced ER stress levels. E. Nuclear translocation of the ER stress downstream molecule ATF4 after SSU72 knockdown was assessed by nuclear-cytoplasmic fractionation assay. F. After GSK2606414 treatment, detection of SSU72 knockdown-induced apoptosis and autophagy markers levels by Western blot.

To functionally confirm this mechanism, we treated control and sh-SSU72 HCT8/HCT116 cells with 2 mM 4-phenylbutyric acid (4-PBA), a chemical chaperone that alleviates ER stress by assisting in the refolding of misfolded proteins, for 36 h^13^. Post-treatment analysis revealed the reduced expression of p-PERK, p-eIF2α, and BIP (Figure 4A), demonstrating that 4-PBA rescued the activation of ER stress in sh-SSU72 cells. Collectively, these experiments demonstrated that SSU72 modulates the PERK-eIF2α axis, a canonical ER stress pathway, to induce malignant phenotypic transformation in CRC. As previously noted, SSU72 functions as a phosphatase critical for transcriptional regulation. Thus, we focused on the relationship between its dephosphorylation activity and ER stress. Calnexin, a lectin-like chaperone localized to the ER membrane^14^, was found to co-localize with SSU72 via immunofluorescence assays (Figure 4B), suggesting a potential interaction with key ER membrane components. Among the three ER transmembrane sensors, namely, IRE1, PERK, and ATF6^15^, prior experiments had established that SSU72 selectively modulates the PERK-eIF2α pathway. To confirm direct interaction, we performed Co-IP assays in HCT-8 cells, demonstrating a physical association between SSU72 and PERK (Figure 4C). To confirm the dependency of SSU72-mediated ER stress on the PERK pathway, we treated CRC cells with 2 μM GSK2606414, a PERK inhibitor, for 48 h. The inhibitor effectively suppressed p-PERK and p-eIF2α and reduced the expression of the chaperone BIP (Figure 4D). ATF4, a key transcription factor in the PERK-eIF2α signaling axis, typically translocates from the cytoplasm to the nucleus under ER stress. Therefore, we examined ATF4 nuclear translocation and observed a marked increase in sh-SSU72 cells (Figure 4E), indicating activation of the PERK/eIF2α/ATF4 cascade. To confirm pathway dependency, we treated cells with the PERK inhibitor GSK2606414 and analyzed apoptosis- and autophagy-associated proteins. SSU72 knockdown in CRC cells combined with GSK2606414 significantly reduced the levels of cleaved caspase-3, cleaved caspase-9, ATG5, and the LC3-II/LC3-I ratio (Figure 4F). Similarly, we examined the expression levels of p-PERK and p-eIF2α by Western blot in SSU72-overexpressing and sh-SSU72# cell lines, and observed results consistent with the above findings (Supplementary Figure 4M&N). These results demonstrate that SSU72 deficiency activates ER stress primarily through the PERK signaling axis, thereby promoting autophagy and apoptosis.

### SSU72 suppresses ER stress in CRC by dephosphorylating the *Thr982* residue of PERK

**Figure 5:**
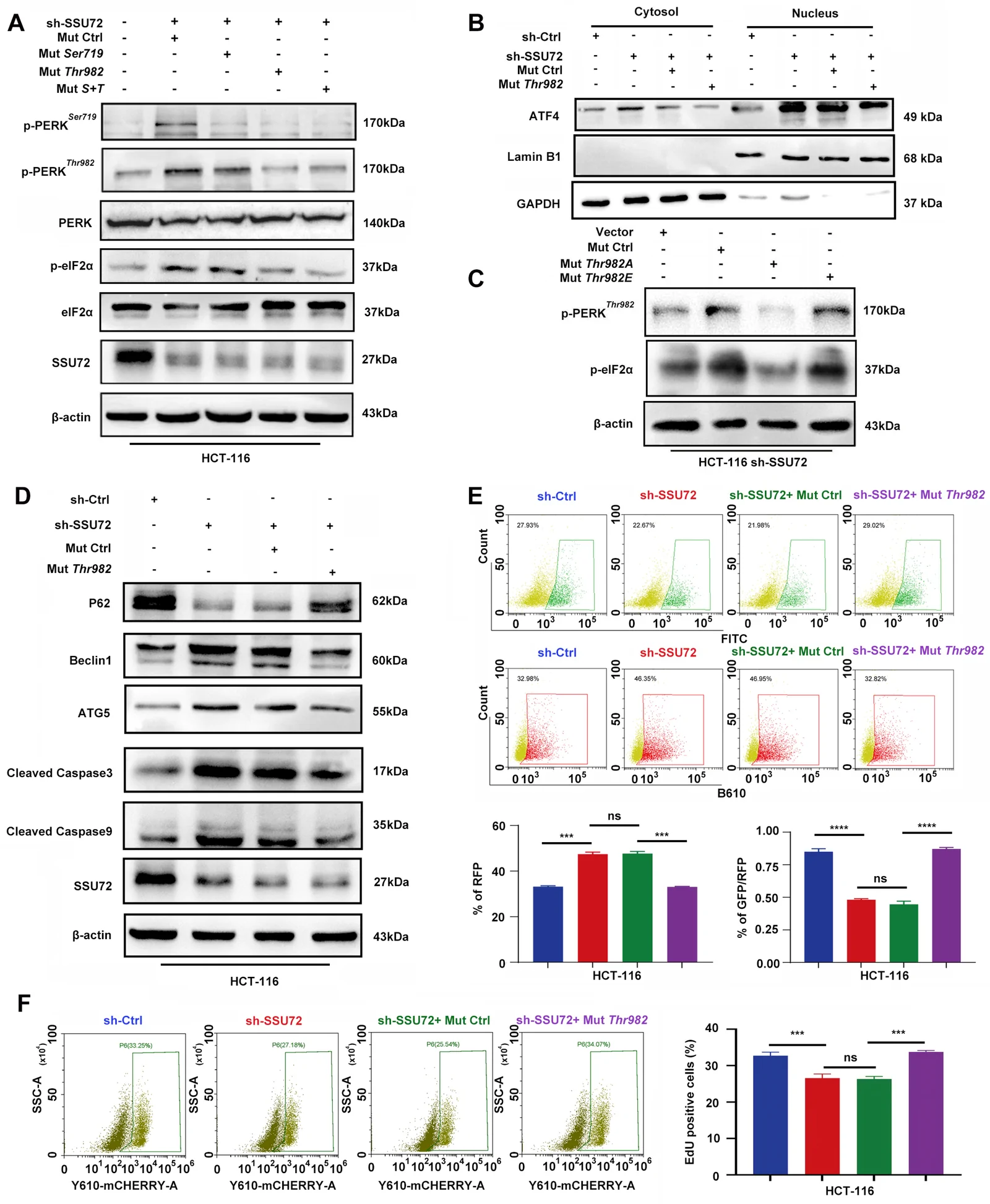
** SSU72 suppresses ER stress in CRC by dephosphorylating the *Thr982* residue of PERK.** A. Mutations at *Thr982*, *Ser719*, and *S719/T982* impaired the ER stress response, as detected by changes in p-PERK and p-eIF2α levels, identifying the critical site for SSU72 binding and phosphorylation regulation of PERK. B. Nuclear translocation of ATF4 mediated by SSU72 knockdown after Thr982 mutation via nuclear-cytoplasmic fractionation assay. C. In HCT-116 sh-SSU72 cells, PERK*^Thr982A^*/*^Thr982E^* mutants were respectively expressed, and the resulting changes in PERK signaling pathway expression were examined. D. Levels of autophagy and apoptosis markers mediated by SSU72 knockdown following *Thr982* mutation by Western blot. E. GFP/RFP ratio-based autophagic flux assay. F. EdU apoptosis analysis further validated the functional consequences of *Thr982* mutation.

Although our preliminary data confirmed that SSU72 directly interacted with PERK, the specific phosphosites through which the phosphatase SSU72 binds to and regulates PERK remain unknown. The literature suggested that *Ser719* and *Thr982* on PERK are critical for its function^16^, ^17^. To investigate whether these sites are involved, we employed site-directed mutagenesis, substituting *serine 719* and *threonine 982* with *alanine (A)* to create phospho-deficient mutants. In collaboration with Shanghai Genechem Co., Ltd., we successfully generated and sequence-verified the following constructs: wild-type PERK (Supplementary Figure 5A), *S719A* (Supplementary Figure 5B), *T982A* (Supplementary Figure 6A), *S719A/T982A* (Supplementary Figure 6B), and corresponding empty vectors (all based on NM_004836.7). To investigate the functional site, we transiently transfected the respective plasmids into sh-SSU72 HCT-116 cells, with sh-Ctrl cells as a control. The results revealed that mutation at the *Thr982 (T982* and *S719/T982)* site impaired the ER stress response, as indicated by the levels of p-PERK and p-eIF2α, whereas mutation at *Ser719* had no significant effect (Figure 5A). These findings suggested that *Thr982* is the specific site through which SSU72 binds to PERK and regulates its phosphorylation.

Subsequently, we examined the nuclear translocation of ATF4 and the levels of downstream autophagy and apoptosis markers following the expression of the *Thr982* mutant. The results showed that the *Thr982* mutation markedly reduced ATF4 nuclear accumulation (Figure 5B). Further validation using functional mutants revealed that expression of the phosphorylation deficient mutant *Thr982A* in sh-SSU72 cells significantly reduced PERK*^Thr982^* phosphorylation levels and suppressed downstream p-eIF2α expression. In contrast, expression of the phosphomimetic mutant* Thr982E* (Supplementary Figure 7A) maintained sustained activation of PERK signaling (Figure 5C). These findings indicate that the phosphorylation status of PERK*^Thr982^* plays a critical role in SSU72 mediated regulation of ER stress signaling. Consequently, the levels of key autophagy markers (P62, Beclin1, and ATG5) and apoptosis markers (cleaved caspase-3/9) were suppressed (Figure 5D). Collectively, these data indicated that mutating the *Thr982* site reverses the sh-SSU72-induced activation of ER stress-mediated ATF4 nuclear translocation, thereby inhibiting subsequent apoptosis. To further validate these functional outcomes, Figure 5E evaluates autophagic flux by measuring changes in the GFP/RFP ratio in an autophagic flux assay. A decrease in the GFP/RFP ratio indicates GFP quenching and unimpaired autophagic flux; this assay quantifies autophagic flux and improves experimental accuracy. We also performed EdU incorporation assays and apoptosis analysis via flow cytometry (Figure 5F). The results consistently confirmed that SSU72 depletion promoted apoptosis. These findings demonstrated that SSU72 deficiency induces autophagy and apoptosis in CRC cells via the PERK*^Thr982^*-eIF2α-ATF4 axis, thereby inhibiting malignant phenotypic transformation.

### SSU72 is a key molecule promoting the malignant phenotypic transformation of CRC by *in vivo* experiments

**Figure 6:**
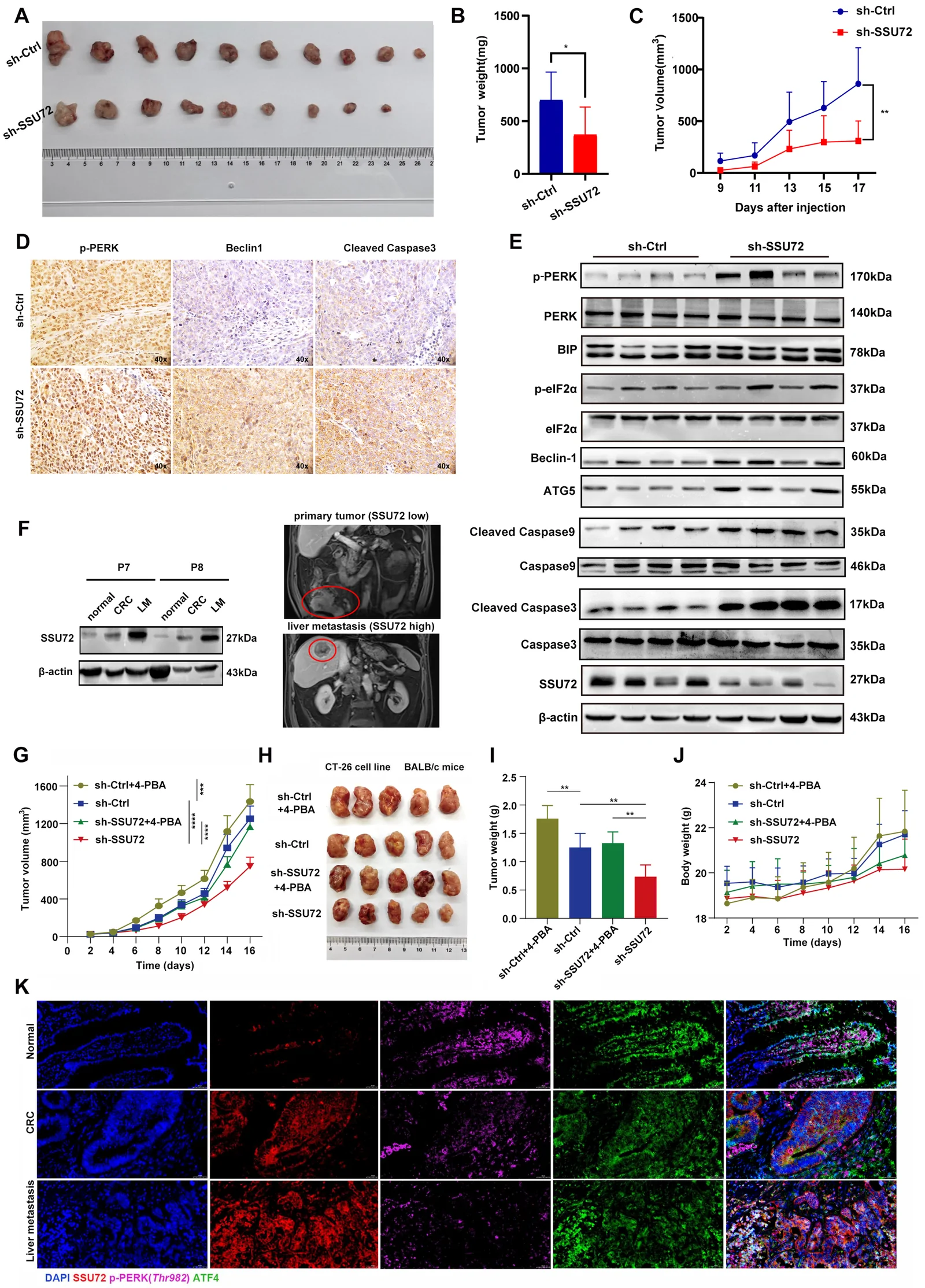
** SSU72 is a key molecule promoting the malignant phenotypic transformation of CRC by* in vivo* experiments.** A. Representative tumor images from the HCT116 sh-Ctrl and sh-SSU72 groups in nude mice. B. Comparison of tumor weight between the two groups. C. Tumor growth curves of the two groups. D. IHC analysis of ER stress, autophagy, and apoptosis markers in xenograft tumors from each group. E. Western blot detection of ER stress, autophagy, and apoptosis markers in mouse tumor tissues from both groups. F. Western blot analysis of SSU72 expression in matched human adjacent normal tissues, primary CRC, and metastatic lesions, accompanied with MR images from a representative patient with colorectal liver metastasis. G-I. CT-26 cell lines with varying SSU72 expression levels and subcutaneously implanted into BALB/c mice to establish xenograft tumors, treated with 4-PBA. J. Tumor size and weight were evaluated, and mouse body weight was also assessed. K. Multiplex immunofluorescence analysis of changes in ER stress signals during the progression of CRC.

We established the nude mouse xenograft model by subcutaneously inoculating HCT116 sh-Ctrl and sh-SSU72 cells. After tumor formation, measurements revealed that the SSU72 knockdown group exhibited significantly reduced tumor weight (Figure 6A&B) and volume (Figure 6C) compared with the control group, indicating markedly diminished malignant potential. IHC analysis of tumor tissues from the two xenograft models demonstrated markedly elevated expression levels of p-PERK, Beclin1, and cleaved caspase3 in the sh-SSU72 group (Figure 6D). Furthermore, Western blot analysis of tumor tissues demonstrated elevated levels of p-PERK and p-eIF2α in the sh-SSU72 group, whereas total PERK and eIF2α levels remained unchanged. Simultaneously, upregulated expression of autophagy-related markers Beclin-1 and ATG5 and increased levels of apoptosis-associated cleaved caspase-3/9 were observed in SSU72-deficient tumors (Figure 6E). To further validate the association between SSU72 and CRC malignancy, we analyzed SSU72 expression in normal intestinal epithelium, primary CRC tissues, and synchronous liver metastases from two patients. A progressive elevation of SSU72 expression was detected with advancing malignant potential (Figure 6F). Representative MR images of P8's primary CRC and liver metastasis are displayed in Figure 6F. We also used immunocompetent BALB/c mice as the model animal and further performed *in vivo* experiments with the CT-26 mouse cell line, using the ER stress inhibitor 4-PBA for conditional intervention. The results showed that, under conditions where 4-PBA intervention did not affect mouse body weight or survival status (Figure 6J), reduced SSU72 expression led to decreased tumor volume, and the addition of 4-PBA reversed the phenotype caused by SSU72 downregulation (Figure 6G-I).

We performed multiplex immunofluorescence staining to assess the expression levels and subcellular localization of SSU72, p-PERK*^Thr982^*, and ATF4 in normal colon tissue, primary tumor tissue, and liver metastatic lesions derived from the same patient with CRC. The results showed that in normal colonic mucosa, SSU72 was weakly expressed, whereas p-PERK*^Thr982^* signals were robust, and ATF4 was predominantly localized in the nucleus, indicating a relatively active ER stress pathway. In primary CRC tissues, SSU72 expression was markedly increased, accompanied by a significant decrease in p-PERK*^Thr982^* signals. Meanwhile, ATF4 translocated from the nucleus to the cytoplasm, suggesting inhibition of the PERK-ATF4 signaling pathway. This trend was further accentuated in liver metastases (Figure 6K).

**Figure 7:**
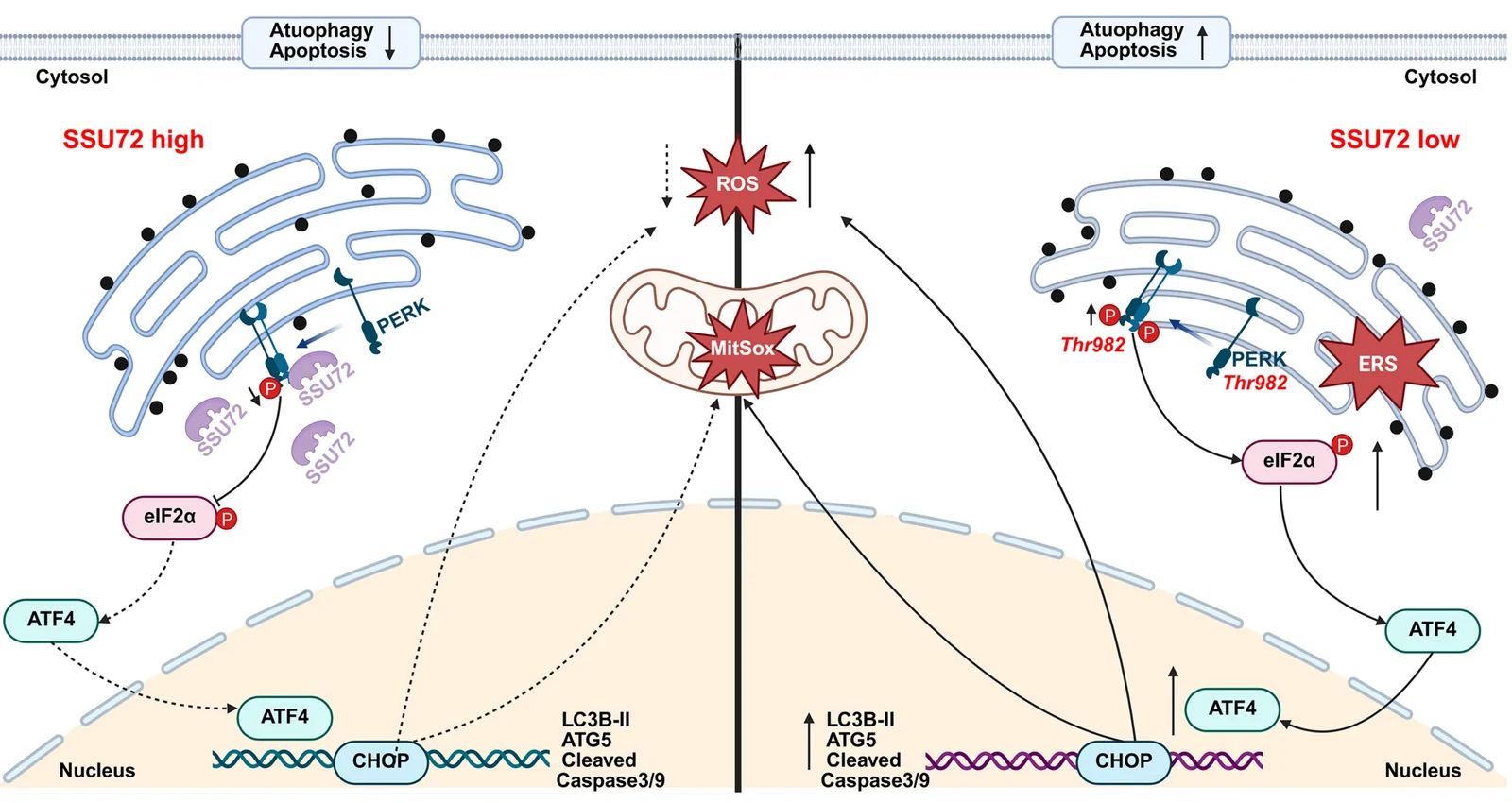
** Schematic illustrating the proposed mechanistic model of this study**.

On the basis of these experimental observations, we preliminarily identified that phosphatase SSU72 interacts with PERK and dephosphorylates it at the *Thr982* site. Through the PERK-eIF2α signaling pathway, SSU72 downregulates ER stress levels in CRC, thereby suppressing the nuclear translocation of ATF4 and mediating reduced levels of tumor cell autophagy and apoptosis, facilitating the malignant progression of CRC (Figure 7).

## Discussion

Globally, CRC is the third most common malignancy and the second leading cause of cancer-related death, accounting for ~1.93 million new cases and ~935,000 deaths in 2020^1^. Conventional chemotherapy achieves an objective response rate (ORR) of 30%-50% in advanced CRC cases, with inherent susceptibility to drug resistance^5^. Targeted therapies demonstrate efficacy in molecularly defined subgroups but are constrained by limited patient eligibility and an absence of viable salvage therapies post-resistance^3^. These challenges highlight the critical need to identify novel molecular biomarkers for the advancement of precision therapies.

To address this clinical challenge, we performed iTRAQ-based proteomic sequencing on tumor tissues from 15 patients with CRC. The cohort was stratified into three groups (n=5 per group) for paired proteomic comparisons, a method that minimized sample bias and ensured robust biological replication. For protein screening, candidate molecules were prioritized based on high FC (>2), selecting the top 20 differentially expressed proteins between groups. Subsequent dual validation using large-scale databases identified the phosphatase SSU72 as the most clinically relevant target. SSU72, a dual-specificity phosphatase (DUSP), has attracted significant interest because of its enzymatic capacity to dephosphorylate serine/threonine (Ser/Thr) and tyrosine (Tyr) residues^8^. Notably, SSU72 modulates RNA transcription initiation through direct interaction with the transcription factor TFIIB^18^. Emerging evidence further reveals its pleiotropic roles beyond transcriptional regulation, including participation in cell cycle progression, immune modulation, hepatic homeostasis, and T-cell activation^7^, ^19^-^21^. Current research on SSU72 in malignancies is scarce, with only two studies reported to date^22^, ^23^. These findings collectively highlight the functional versatility of SSU72 beyond its canonical phosphatase activity. Our focus on elucidating the phosphatase-dependent mechanisms by which SSU72 regulates phenotypic and molecular alterations in CRC represents a novel conceptual advancement in this field.

Our experimental approach integrating apoptosis assays, autophagic flux evaluation, TEM, and Western blot analysis revealed a critical association between SSU72 and autophagy/apoptosis regulation in CRC. Notably, prior studies demonstrated that USP33 overexpression in prostate cancer induces docetaxel resistance by suppressing the degradation of its binding partner DUSP1, thereby attenuating JNK activation and apoptosis^24^. This mechanism exhibits striking parallels with our findings on SSU72-mediated apoptotic suppression in CRC, suggesting conserved regulatory roles among DUSP family members in apoptosis evasion. The concurrent observation of apoptotic bodies and autophagic vesicles prompted our investigation into the dual regulatory role of SSU72 in tumor autophagy. TMT-based proteomic sequencing further localized SSU72's functional activity primarily to ER-associated compartments, with ER stress emerging as its principal mechanistic pathway. This corresponds with prior studies establishing the ER-autophagy connection. Under nutrient stress, mammalian cells dynamically remodel their proteome through transcriptional, translational, and degradative adaptations^25^. Emerging evidence indicates that multiple transmembrane ER proteins act as receptors to recruit core autophagy components for autophagosome formation^26^, ^27^, a process mechanistically associated with SSU72-mediated ER stress modulation in our experimental system.

As previously established, the UPR constitutes the core mechanism of ER stress^28^. The UPR restores proteostatic equilibrium through three major signaling branches: PERK-eIF2α-ATF4, IRE1-XBP1, and ATF6 translocates to the Golgi for proteolytic cleavage^29^, ^30^. RT-PCR analysis of SSU72-modulated pathways in CRC ER stress models identified predominant dysregulation within the PERK-eIF2α axis. Immunofluorescence co-localization revealed a spatial association between SSU72 and the ER membrane marker calnexin, suggesting interaction with ER transmembrane components. Given the three canonical ER stress sensors (IRE1, PERK, and ATF6)^15^ and our prior evidence of SSU72's selective PERK-eIF2α pathway engagement, CO-IP assays in HCT-8 CRC cells confirmed direct SSU72-PERK interaction. This mechanistic clarification establishes PERK as the primary molecular target through which SSU72 modulates ER stress in CRC. Various physicochemical stimuli lead to the accumulation of unfolded proteins, thereby activating the IRE1, PERK, and ATF6 signaling pathways^31^, ^32^. In our study, we primarily focused on the PERK/eIF2α/ATF4 signaling axis following SSU72 dysregulation. This pathway is known to upregulate key autophagy-related genes and also trigger CHOP mediated apoptosis^33^, ^34^. Our recent evidence suggests that SSU72 may modulate the PERK/eIF2α signaling axis by regulating protein phosphatase activity. Specifically, SSU72 deficiency induces autophagy (increased ATG5 and LC3-II levels) in an eIF2α phosphorylation dependent manner. In contrast, prolonged SSU72 deficiency leads to predominant activation of CHOP and caspase-3, resulting in elevated apoptosis. Autophagy likely serves as a buffer between SSU72 dysregulation and apoptosis, a biological axis that warrants further validation in future studies.

Given the intrinsic phosphatase activity of SSU72, precise identification of its dephosphorylation site(s) on PERK is critical to elucidate its functional specificity. Literature mining revealed two established phosphorylation sites on PERK: *Ser719*^35^ and *Thr982*^17^, ^36^. To identify the target residue, we generated phosphorylation-null mutant plasmids by substituting *Ser719* and *Thr982* with alanine (Ala) via site-directed mutagenesis, thereby ablating their phosphorylation capacity^37^. Western blot analysis demonstrated that SSU72 specifically dephosphorylated PERK at *Thr982*. This finding possesses mechanistic significance, as PERK's primary role in ER stress and UPR activation is the translational inhibition of nascent proteins. Phosphorylation at *Thr982* is a well-established biomarker of ER stress, where activate PERK phosphorylates eIF2α to decrease global protein synthesis, thereby restoring proteostatic homeostasis^38^, ^39^. Our data confirmed that *Thr982* was the critical regulatory node through which SSU72 modulated PERK-mediated translational control under ER stress.

The traditional perspective posits that sustained and severe ER stress exerts tumor-suppressive effects by triggering irreversible apoptotic signaling^28^. However, recent studies have revealed a complex scenario: a moderate or context-specific activation of the UPR, particularly certain signaling pathways, is not only non-lethal but can be utilized by tumor cells to enhance survival, adapt to hostile microenvironments, and develop therapeutic resistance^15^. This shift from a "pro-death" to a "pro-survival" role represents a central paradox in ER stress biology and poses a major challenge for therapeutic intervention^29^. Within this complex regulatory network, our study identified SSU72 as a critical player. Beyond its fundamental transcriptional regulatory functions, SSU72 acts as a key molecular node that may directly influence the balance and transduction of UPR branch signals.

Notably, in normal cells, mild to moderate ER stress restores homeostasis through the UPR and is thus protective^40^. In contrast, in already transformed tumor cells, ER stress is often chronically activated due to metabolic pressure and microenvironmental hypoxia^41^. Under such conditions, partial inhibition of ER stress signaling (SSU72 modulation) may selectively reduce the apoptotic burden of tumor cells, thereby conferring a growth advantage. The 'pro-tumorigenic effect of ER stress dysregulation' observed in our study is therefore context dependent, occurring under specific tumor cell models and SSU72 intervention conditions. Furthermore, SSU72 primarily affects the PERK/eIF2α/ATF4 branch rather than the IRE1 or ATF6 branches. Different branches can produce distinct or even opposing functional outcomes^42^. For example, the PERK branch can simultaneously initiate both autophagy and apoptosis^34^, ^43^, whereas the IRE1 branch regulates inflammation and cell survival^44^. Similarly, most studies support an intensity dependent model of ER stress^45^: chronic or excessive ER stress can trigger apoptosis and thereby suppress further tumor growth^46^. Our findings align with this model, positioning SSU72 as a regulator that tilts the balance toward pro-survival UPR output under chronic stress conditions, ultimately accelerating malignant progression.

*In vivo* experiments further confirmed that SSU72 enhances the malignant potential of CRC. This phenomenon was consistently observed in various tissues from patients with CRC and synchronous liver metastasis through SSU72 expression level detection, and these findings were validated by single-cell sequencing data. These results collectively suggested that elevated SSU72 expression not only promotes tumor growth but also enhances the potential for distant metastasis. As previously mentioned, metastatic CRC demonstrates low response rates to chemotherapy and has few targeted therapeutic options. Therefore, SSU72 may serve as a novel therapeutic target for CRC-targeted treatment and efficacy prediction to improve patient prognosis, although this requires validation through clinical trials.

This study has several limitations. In the initial iTRAQ sequencing, multiple patient samples were pooled for proteomic analysis, which may have, to some extent, reduced the representation of interindividual heterogeneity. This is a limitation of the discovery phase of our study. PERK is a large protein containing multiple transmembrane domains and a complex intracellular kinase domain, obtaining high-purity, correctly folded, and phosphorylation-maintained full-length PERK protein is extremely challenging. This limits the feasibility of purifying PERK protein for biochemical assays. These issues will be further addressed in future studies.

## Supplementary Material

Supplementary materials and methods, figures.

Supplementary table 1: Primer sequences used in this study.

Supplementary table 2: iTRAQ-based proteomic sequencing.

Supplementary table 3: ER stress gene list.

## Abbreviations

CRC: colorectal cancer
ER: endoplasmic reticulum
GO: Gene Ontology
CQ: chloroquine
TMT: Tandem Mass Tag
TCGA: The Cancer Genome Atlas
KEGG: Kyoto Encyclopedia of Genes and Genomes
PCA: Principal component analysis
p-PERK: phosphorylated PERK
p-eIF2α: phosphorylated eIF2α
4-PBA: 4-phenylbutyric acid
ROS: reactive oxygen species
Mito SOX: mitochondrial ROS
ORR: objective response rate
DUSP: dual-specificity phosphatase
Ser: serine
Thr: threonine
Tyr: tyrosine
TEM: transmission electron microscopy
DEGs: differentially expressed genes
Co-IP: co-immunoprecipitation
