# Supplementary material for: Novel Phosphatase SSU72 Drives Malignant Progression in Colorectal Cancer via PERKThr982 Dephosphorylation Mediated Endoplasmic Reticulum Stress Inactivation
Source: Int J Biol Sci. 2026 Jul 22;22(13):6876–91. doi: 10.7150/ijbs.129555 (PMC13540442; doi:10.7150/ijbs.129555)
Supplement: Supplementary file 1 [file ijbsv22p6876s1.pdf]

Materials and Methods

1.1 Western blot and antibodies

The following antibodies were used: **SSU72**, Cell Signaling Technology, #12816, 1:1000. **β-actin**, Santa Cruz, #sc-47778, 1:1000. **caspase-3**, Cell Signaling Technology, #9662, 1:1000. **caspase-9/cleaved caspase-9**, Proteintech, #10380-1-AP, 1:1000. **cleaved caspase-3**, Cell Signaling Technology, #9661, 1:1000. **ATG5**, Cell Signaling Technology, #12994, 1:1000. **Beclin1**, Cell Signaling Technology, #3495, 1:1000. SQSTM1/p62, Cell Signaling Technology, #23214， 1:1000. **LC3B**, Cell Signaling Technology, #3868, 1:1000. **PERK**, ABclonal, #A18196, 1:1000. **Phospho-PERK**^{Ser719}, Proteintech, #29546-1-AP, 1:1000. **Phospho-PERK**^{Thr982} Rabbit pAb, ABclonal, #AP0886, 1:1000. **BIP**, Cell Signaling Technology, #3177, 1:1000. **eIF2α**, Cell Signaling Technology, #5324, 1:1000. **Phospho-eIF2α**, Cell Signaling Technology, #3398, 1:1000. **ATF6**, zenbio, #500202, 1:1000. ATF6, Cell Signaling Technology, #65880, 1:1000. **Phospho-IRE1**^{Ser724}, zenbio, #530878, 1:1000. **ATF4**, SAB, #49147, 1:1000. **m-IgG-BP-HRP**, Santa Cruz, #sc-516102, 1:4000. **mouse anti-rabbit IgG-HRP**, Santa Cruz, #sc-2357, 1:4000.

Freshly resected CRC specimens and paired adjacent non-tumor tissues were homogenized in liquid nitrogen and lysed using a tissue protein extractor (Servicebio). Cells were washed three times with cold PBS (pH 6.8) and lysed in ice-cold SDS buffer containing 1 mM NaF, 1 mM Na_3VO_4, and 1×protease/phosphatase inhibitor cocktail (Roche) for 30min. Lysates were denatured at 95°C for 10 min, centrifuged (12,000 rpm, 4°C, 10min), and resolved by SDS–PAGE (30–60 μg/lane). Proteins were transferred to PVDF membranes (Immobilon-P, Millipore), blocked with 5% non-fat milk or 3% BSA, and probed with primary antibodies at 4°C overnight. Membranes were incubated with HRP-conjugated secondary antibodies (1:4,000) for 1 h at room temperature (RT).

1.2 RT-qPCR and primer sequences

Total RNA was isolated from adherent cells by using TRIzol reagent (Ambion). cDNA was synthesized from 1 μg of RNA using a PrimeScript RT-qPCR kit (Takara) according to the manufacturer's protocol. For mRNA quantification, RT-qPCR amplification was performed with predesigned primers (Takara) under standard cycling conditions. β-Actin served as an endogenous

control for mRNA. Primer sequences are listed in Supplementary Table 1.

1.3 CCK-8 assay

Cells in the logarithmic growth phase were harvested by trypsinization, centrifuged, and counted. Cell suspensions were adjusted to 2×10^3 cells/100 μL in complete medium and seeded into 96-well plates (six replicate wells per group). After 6 h of incubation (37°C, 5% CO_2), 10 μL of Cell Counting Kit-8 (CCK-8, Dojindo Molecular Technologies, Japan) was added to each well. Following 2.5h of incubation, absorbance at 450 nm was measured using a Synergy H1 microplate reader (BioTek, USA), designated as Day0. Subsequent measurements were performed daily under identical conditions from Day 1 to Day 5.

1.4 EdU proliferation assay

Cells were seeded in 6-well plates at 3×10^5 cells/well and incubated at 37°C under 5% CO_2. At 65%–75% confluency, cells were pulsed with 1× EdU working solution (pre-warmed to 37°C for 15min) diluted 1:1 in culture medium for 2.5 h. After EdU labeling, cells were fixed with 4% paraformaldehyde (1 mL/well, 15 min at RT), washed three times with PBS containing 3% BSA (3–5min/wash), and permeabilized with 0.3% Triton X-100 in PBS (1 mL/well, 10–15min at RT). Following permeabilization, cells were incubated with Click reaction cocktail (0.5 mL/well, prepared per manufacturer's protocol) for 30 min at RT in the dark. After three washes (3–5min/wash), nuclei were counterstained with Hoechst 33342 (1:1000 in PBS, 1mL/well, 10min at RT).

1.5 Apoptosis analysis

Cells were washed in ice-cold PBS followed by 200 μL of 1 x binding buffer and then stained for 15 minutes in the dark with Annexin V-APC and 7-Amino-Actinomycin (7-AAD) at room temperature (BD Pharmingen™, 559763). Herein, 400-800 μL of 1× binding buffer was added based on the number of cells, and then the cells were analyzed. Then, the percentage of apoptotic cells was analyzed by the Beckman Coulter flow cytometer.

1.6 Immunofluorescence staining

Cells were seeded on glass coverslips in 12-well plates (1×10^5 cells/well) and cultured for 36 h. After discarding the medium, cells were washed with PBS and fixed with 4% paraformaldehyde (PFA;

10 min at RT). Following two PBS washes, cells were permeabilized with 0.1% Triton X-100 in PBS (15min). Primary antibodies diluted in antibody buffer included anti-SSU72 (1:100; Cell Signaling Technology, #12816S) and anti-Calnexin (1:50; Proteintech, #66903-1-Ig). Coverslips were incubated with primary antibodies overnight at 4°C. After PBS washes, coverslips were incubated with fluorescence-conjugated secondary antibodies (1:500; species-matched) for 1h at RT. Nuclei were counterstained with DAPI (5 μg/mL; 10 min), and images were acquired using a fluorescence microscope.

1.7 Transmission electron microscopy (TEM)

TEM was conducted according to the manufacturer's instructions.

1.8 Autophagic flux assay

The mRFP-GFP-LC3 dual fluorescence system was employed to assess autophagic activity. HCT-116 and HCT-8 cells were seeded in 6-well plates at 1×10^5 cells/well and allowed to adhere. After morphological recovery, cells were transfected with stubRFP-sensGFP-LC3 Lentivirus (GPL2001, Gene, Shanghai, China) and cultured for 72h under standard conditions (37°C, 5% CO_2). GFP (green fluorescence) and mRFP (red fluorescence) signals were visualized using a confocal microscope. GFP labels autophagosomes, whereas mRFP labels autolysosomes. Differences in red fluorescence indicate the intensity of autophagic flux. The green-to-red ratio should reflect the smoothness of autophagic flux.

1.9 Nuclear–cytoplasmic fractionation

The cytoplasmic and nuclear proteins were extracted using a nuclear and cytoplasmic extraction kit (Thermo, 78835). All procedures were performed on ice. CERI, CERII, and NER reagents were supplemented with 50× Protease Inhibition Cocktail prior to use. Approximately 2×10^6 cells per sample were collected, trypsinized, and centrifuged at 1,000 rpm for 5 min at 4°C. After the supernatant was completely removed, the cells were washed once with PBS and centrifuged again under the same conditions. The resulting cell pellet was mixed with 100 μl of CERI, vortexed to resuspend, and incubated on ice for 10 min. Then, 5.5 μl of ice-cold CERII was added, followed by vortexing and incubation on ice for 1 min. After another vortex for 10-15 s, the mixture was

centrifuged at 16,000 × g for 20 min at 4°C. The supernatant, representing the cytoplasmic protein fraction, was transferred to a new EP tube and stored at -80°C. The remaining pellet was resuspended in 50 µl of NER, vortexed, and incubated on ice for 40 min with vortexing every 10 min. Following a final vortex for 10-15 s, the sample was centrifuged at 16,000 × g for 40 min at 4°C. The supernatant, representing the nuclear protein fraction, was transferred to a new EP tube and stored at -80°C.

1.10 Co-IP

Co-IP was performed using an immunoprecipitation kit (Yamei, #YJ201) following the manufacturer's protocol. Cells were lysed in ice-cold IP lysis buffer supplemented with protease and phosphatase inhibitors. Lysates were centrifuged at 12,000 × g for 10 min (4°C), and supernatants were incubated with anti-PERK (Santa Cruz, #sc-377400) or anti-SSU72 antibodies for 1 h at RT to form antigen–antibody complexes. Pre-washed Protein A/G magnetic beads were added to the lysate–antibody mixtures and incubated for 2 h at RT. Beads were then washed five times with lysis buffer, and bound proteins were eluted in SDS loading buffer. Eluted proteins were resolved by SDS–PAGE, transferred to PVDF membranes, and probed with primary antibodies against PERK and SSU72 (1:1,000; #12816S).

1.11 Flow cytometry assay

The cell suspension was transferred into flow tubes at about 1×10^6 cells per tube, which was added with fluorescent labeling antibody. The tubes were incubated at 4℃ for 30 min protected from light and washed with PBS twice to remove unbound antibody. Cells were first fixed with 4% paraformaldehyde for 15 min at RT and centrifuged. After the supernatant was discarded, cells were treated with 0.1% Triton X-100 for 10 min. Thereafter, cells were incubated with intracellular antibody, washed, and resuspended. Negative control and single positive controls were established. The flow cytometer was activated, and the laser was pretreated. A liquid flow system rinse was performed, and the instrument was calibrated. The cell suspension was gently mixed to avoid air bubbles. The acquisition parameters were configured, and the flow rate was adjusted. The data were collected and analyzed.

1.12 Construction of PERK phosphorylation site mutant plasmids

In collaboration with Genechem Company (Shanghai, China), we applied alanine (Ala) to replace serine and threonine in the corresponding sites of *Ser719* and *Thr982* by mutant truncation technology, preventing them from exerting their phosphorylation functions. NM_004836.7(*S719A*), NM_004836.7(*T982A*), NM_004836.7(*S719/T982A*), wild-type, and related empty plasmids were constructed, and the plasmids were validated.

1.13 Xenograft model

Stable sh-SSU72 and control CRC cells were washed three times with PBS, resuspended in sterile PBS, and counted. Each BALB/c nude mouse received a subcutaneous injection of (1–2) $\times 10^6$ cells in 100 μL of PBS using a 1 mL syringe. Body weight and tumor growth were monitored every 48 h. Tumor volume was calculated as (length × width^2)/2 using digital calipers. Mice were housed under SPF conditions with ad libitum access to food/water.

1.14 SSU72 expression analysis

CRC-related transcriptome data were downloaded from The Cancer Genome Atlas (TCGA) database using the TCGAbiolinks package in R. The TCGA data contain gene expression profiles of both tumor and normal tissues. Multiple colorectal cancer-related gene expression microarray datasets were downloaded from the Gene Expression Omnibus (GEO) database using the GEOquery package, including GSE113513, GSE21825, GSE31782, GSE44076, and GSE87211.These datasets contain tumor samples and normal samples used to validate and extend the analysis results of TCGA data. All downloaded data were normalized and quality-controlled, and some low-quality expressed genes were filtered to ensure the accuracy of the analysis. The potential role of SSU72 in colorectal cancer was assessed by comparing its expression level in tumor tissues and normal tissues. Visualization of SSU72 gene expression data was performed using the ggplot2 package (version 3.5.2) in R.

1.15 Single cell sequencing analysis

The CRC single-cell datasets GSE178318, GSE188711, GSE132465 and GSE163974 involved in this project were obtained by downloading from the GEO databas, and the processing and analysis of the single-cell data were done by the Seurat R package (version 4.0.0). First, Seurat objects were constructed using the CreateSeuratObject function. Low-quality cells were removed according to the

following criteria: 500< nFeature_RNA <5000 and the proportion of mitochondrial genes (Percent.mt) < 5%. The data were then normalized using the NormalizeData function and scaled using the ScaleData function. In order to explore the differences between cells, the group firstly used the RunPCA function to perform principal component analysis, and selected appropriate principal components based on ElbowPlot and JackStrawPlot. Subsequently, FindNeighbors and FindClusters were used for cell clustering, and the clustering resolution was set according to the number of cells to ensure reasonable cell subpopulation delineation. Canonical marker genes retrieved from the CellMarker 2.0 database were used for cell-type annotation[10]. Finally, dimensionality reduction was performed using UMAP to demonstrate the distribution of different cell populations.

1.16 ER stress gene score and SSU72 gene expression analysis

To assess the level of ER stress in cells, we calculated the score of the ER stress gene set by using the AddModuleScore function. Subsequently, we plotted a correlation point map illustrating the relationship between the expression level of SSU72 and the ER stress score by using the ggplot2 R package. Spearman correlation analysis was conducted to calculate the correlation coefficient and its statistical significance.

1.17 Plasmid information

sh-SSU72 (human)：

oligo1 5'-ccggCCAGAACTGCAAAGACCTGTTctcgagAACAGGTCTTTGCAGTTCTGGttttt-3'.

oligo2 5'-aattaaaaaCCAGAACTGCAAAGACCTGTTctcgagAACAGGTCTTTGCAGTTCTGG -3'.

sh-SSU72# (human):

oligo1 5'-ccggCAGAACTGCAAAGACCTGTTTctcgagAAACAGGTCTTTGCAGTTCTGttttt-3'

oligo2 5'-aattaaaaaCAGAACTGCAAAGACCTGTTTctcgagAAACAGGTCTTTGCAGTTCTG -3'

sh-SSU72 (mouse):

oligo1 5'-ccggCCACCTTTGTTGAAGCCACTTctcgagAAGTGGCTTCAACAAAGGTGGttttt-3'

oligo2 5'-aattaaaaaCCACCTTTGTTGAAGCCACTTctcgagAAGTGGCTTCAACAAAGGTGG-3'

SSU72 overexpression (human):

pcdh-SSU72-Nhel1-F: gagaGCTAGCgccaccGCTAGCATGCCGTCGTCCCC

pcdh-SSU72-Bamh1-R: gagaGGATCCTCAGTAGAAGCAGACGGTGTGC

Supplementary Figure 1

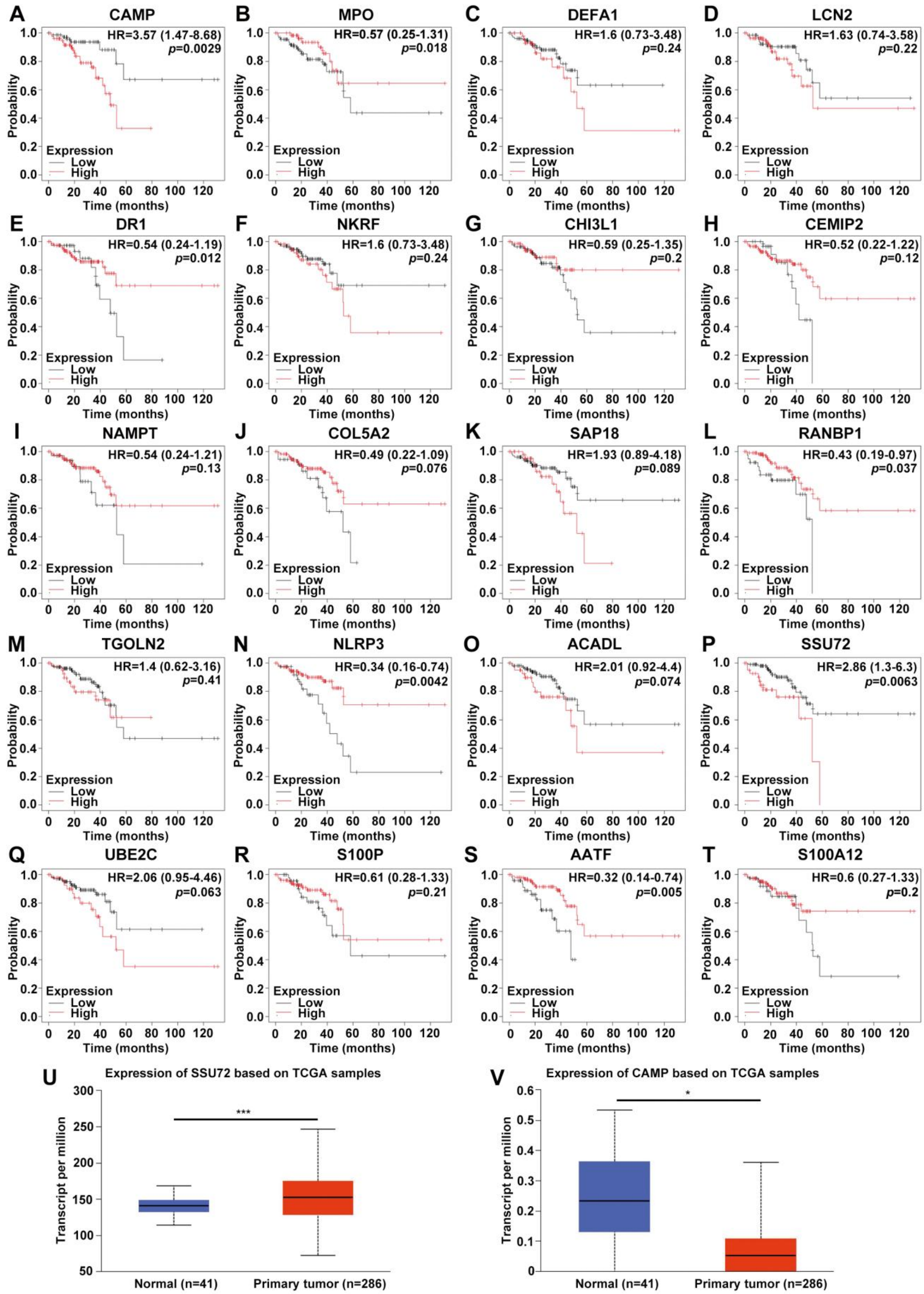

Supplementary Figure 2

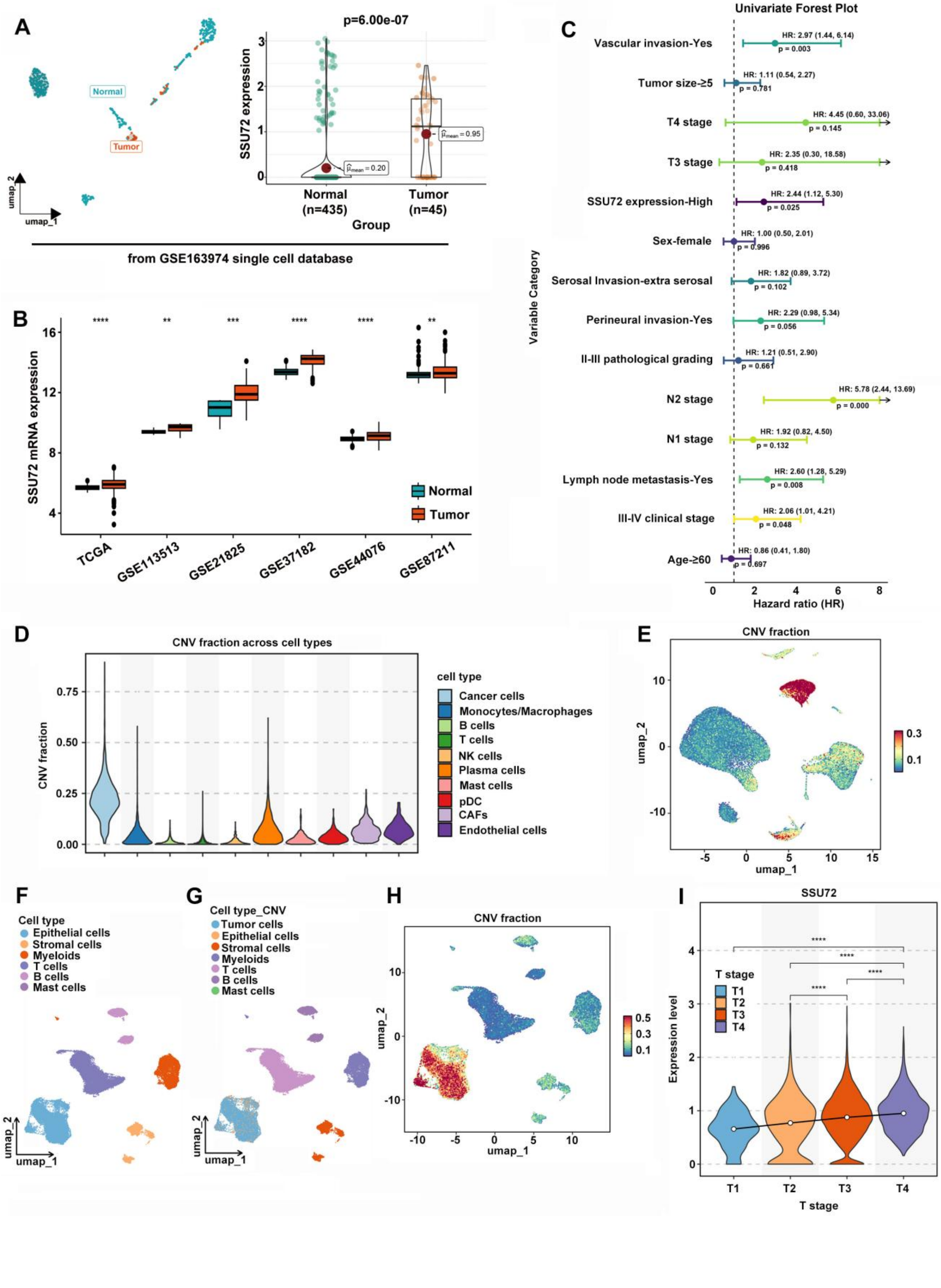

Supplementary Figure 3

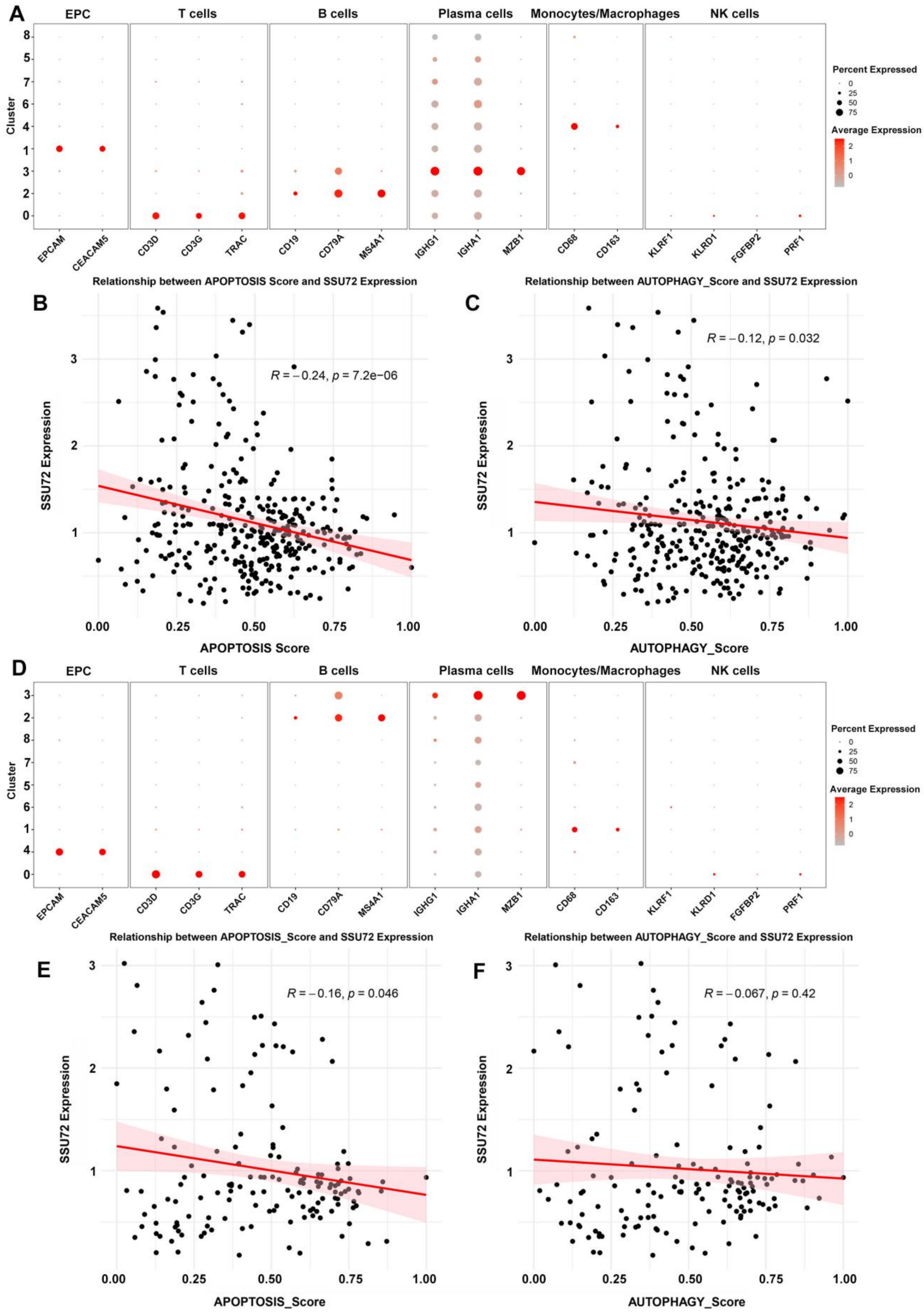

Supplementary Figure 4

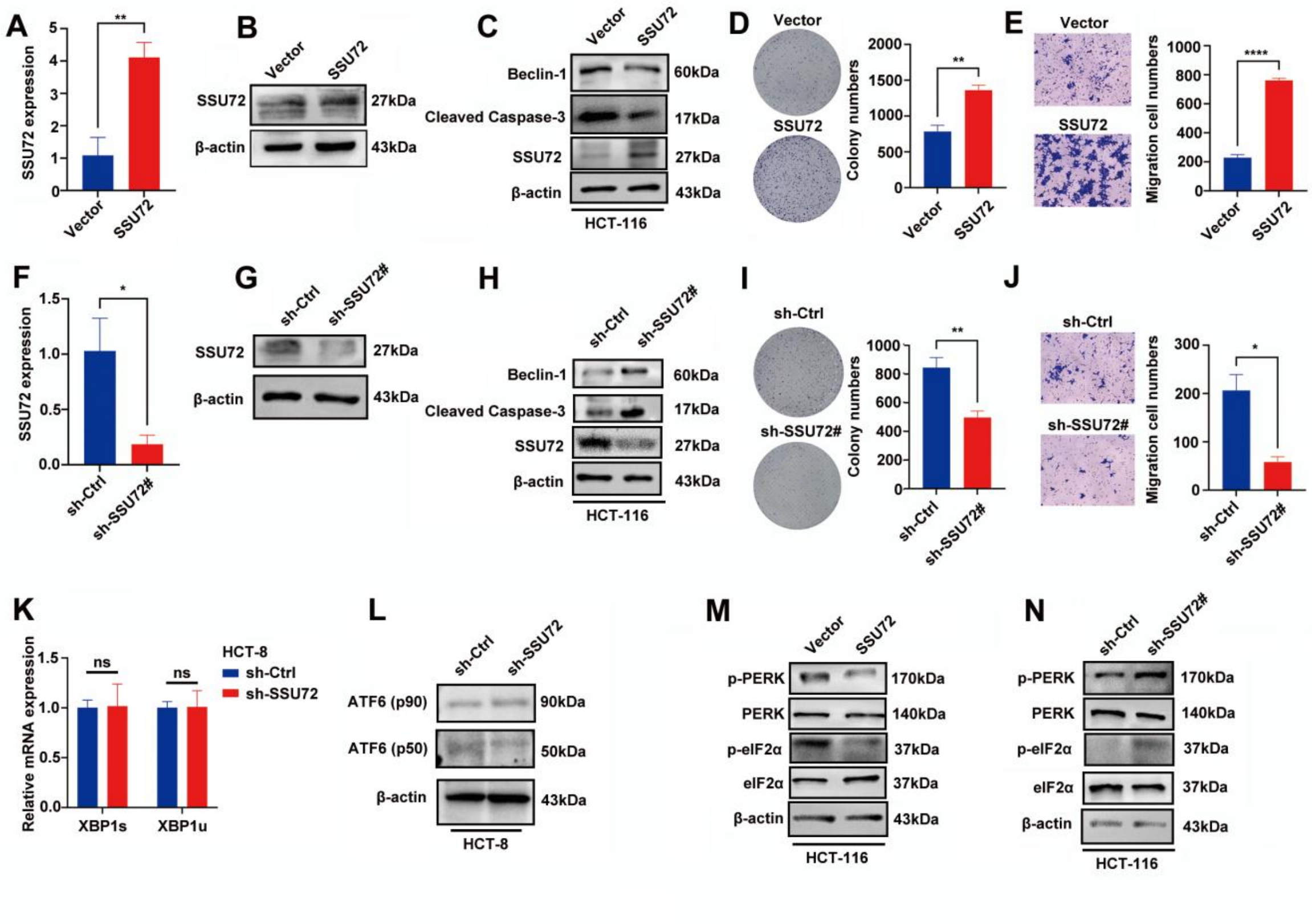

Supplementary Figure 5

A

GENE	Species	GENE_ID	GenBank_ID
PERK/EIF2AK3	Human	9451	NM_004836.7
**Primer Designing for Constructing**		**Primer sequence（5’ - 3’）**	
KL61511-1-P1		GCCCTCTAGACTCGAGCGCCACCATG GAGCGCGCCATCAGCCCGGGGC	
KL61511-1-P2		TAGTCACTTAAGCTTGGTACCGAATTG CTTGGCAAAGGGCTATGGGAG	
**Primer Designing for Identification**		**Primer sequence（5’ - 3’）**	
KL61511-1-F		TTTAAACGGGCCCTCTAGAC	
KL61511-1-R		GCAGCGCCTCAGCGTCCTCC	

Plasmid extraction results:

Label	Batch number	Plasmid concentration (ng/μl)
PL-EIF2AK3(KL61511-1)	134DAE2	386.6

The primers contain homologous arms, restriction sites, and a sequence matching the 5' end of the gene. Primer design schematic for the **3,394-bp** target gene cloning. Colony PCR validation of positive clones produces a **267-bp** fragment.

B

GENE	Species	GENE_ID	GenBank ID
PERK/EIF2AK3	Human	9451	NM_004836.7 (S719A)
**Primer Designing for Constructing**		**Primer sequence（5’ - 3’）**	
K24L1535-P1		ACGGGCCCTCTAGACTCGAGCGCCAC Catggagcgcgccatcagcccggggctg	
K24L1535-P4		AGTCACTTAAGCTTGGTACCGAattgcttg gcaaagggctatgggagttg	
**Primer Designing for Identification**		**Primer sequence（5’ - 3’）**	
K24L1535-wf5		aagtagggaccaaactgtat	
pcDNA-SEQR		TTATTAGGAAAGGACAGTGGG	
**Sequencing Primer**		**Primer sequence（5’ - 3’）**	
CMV-F		CGCAAATGGGCGGTAGGCGTG	
K24L1535-wf1		GATGTTGTTTTGGTTGGAGG	
K24L1535-wf2		agctgtatctgcagtcatca	
K24L1535-wf3		GCCAATTCAATGCCTGGGAC	
K24L1535-wf4		ttgtccttctgaagcttctc	
K24L1535-wf5		aagtagggaccaaactgtat	
pcDNA-SEQR		TTATTAGGAAAGGACAGTGGG	

Plasmid extraction results:

Label	Batch number	Plasmid concentration (ng/μl)
PL-EIF2AK3(K24L1535)	134DAE6	329.3

The primers contain homologous arms, restriction sites, and a sequence matching the 5' end of the gene. Primer design schematic for the **3,394-bp** target gene cloning. Colony PCR validation of positive clones produces a **614-bp** fragment.

Supplementary Figure 6

A

GENE	Species	GENE_ID	GenBank ID
PERK/EIF2AK3	Human	9451	NM_004836.7 (T982A)
**Primer Designing for Constructing**		**Primer sequence（5' - 3'）**	
K24L1537-P1		ACGGGCCCTCTAGACTCGAGCGCCAC Catggagcgcgccatcagcccggggctg	
K24L1537-P6		AGTCACTTAAGCTTGGTACCGAattgcttg gcaaagggctatgggagttg	
**Primer Designing for Identification**		**Primer sequence（5' - 3'）**	
K24L1537-wf5		aagtagggaccaaactgtat	
pcDNA-SEQR		TTATTAGGAAAGGACAGTGGG	
**Sequencing Primer**		**Primer sequence（5' - 3'）**	
CMV-F		CGCAAATGGGCGGTAGGCGTG	
K24L1537-wf1		GATGTTGTTTTGGTTGGAGG	
K24L1537-wf2		agctgtatctgcagtcatca	
K24L1537-wf3		GCCAATTCAATGCCTGGGAC	
K24L1537-wf4		ttgtccttctgaagcttctc	
K24L1537-wf5		aagtagggaccaaactgtat	
pcDNA-SEQR		TTATTAGGAAAGGACAGTGGG	

Plasmid extraction results:

Label	Batch number	Plasmid concentration (ng/μl)
PL-EIF2AK3(K24L1536)	134DAE7	271.3

B

GENE	Species	GENE_ID	GenBank ID
PERK/EIF2AK3	Human	9451	NM_004836.7 (S719/T982A)
**Primer Designing for Constructing**		**Primer sequence（5' - 3'）**	
K24L1536-P1		ACGGGCCCTCTAGACTCGAGCGCCAC Catggagcgcgccatcagcccggggctg	
K24L1536-P6		AGTCACTTAAGCTTGGTACCGAattgcttg gcaaagggctatgggagttg	
**Primer Designing for Identification**		**Primer sequence（5' - 3'）**	
K24L1536-wf5		aagtagggaccaaactgtat	
pcDNA-SEQR		TTATTAGGAAAGGACAGTGGG	
**Sequencing Primer**		**Primer sequence（5' - 3'）**	
CMV-F		CGCAAATGGGCGGTAGGCGTG	
K24L1536-wf1		GATGTTGTTTTGGTTGGAGG	
K24L1536-wf2		agctgtatctgcagtcatca	
K24L1536-wf3		GCCAATTCAATGCCTGGGAC	
K24L1536-wf4		ttgtccttctgaagcttctc	
K24L1536-wf5		aagtagggaccaaactgtat	
pcDNA-SEQR		TTATTAGGAAAGGACAGTGGG	

Plasmid extraction results:

Label	Batch number	Plasmid concentration (ng/μl)
PL-EIF2AK3(K24L1537)	134DAE7	245.2

The primers contain homologous arms, restriction sites, and a sequence matching the 5' end of the gene. Primer design schematic for the **3,394-bp** target gene cloning. Colony PCR validation of positive clones produces a **614-bp** fragment.

Supplementary Figure 7

A

GENE	Species	GENE_ID	GenBank ID
PERK/EIF2AK3	Human	9451	NM_004836.7 (T982E)

Primer Designing for Constructing	Primer sequence （5' - 3'）
K26A0920-P1	ACGGGCCCTCTAGACTCGAGCGCCAC Catggagcgcgccatcagcccggggctg
K26A0920-P4	AGTCACTTAAGCTTGGTACCGAattgcttg gcaaagggctatgggagttg
**Primer Designing for Identification**	**Primer sequence （5' - 3'）**
K26A0920-wf5	aagtagggaccaaactgtat
pcDNA-SEQR	TTATTAGGAAAGGACAGTGGG
**Sequencing Primer**	**Primer sequence （5' - 3'）**
CMV-F	CGCAAATGGGCGGTAGGCGTG
K26A0920-wf1	GATGTTGTTTTGGTTGGAGG
K26A0920-wf2	agctgtatctgcagtcatca
K26A0920-wf3	actgattttgagccaattca
K26A0920-wf4	ttgtccttctgaagcttctc
K26A0920-wf5	aagtagggaccaaactgtat
pcDNA-SEQR	TTATTAGGAAAGGACAGTGGG

Plasmid extraction results:

Label	Batch number	Plasmid concentration (ng/μl)
PL-EIF2AK3(K24L1537)	134DAE7	454.3

The primers contain homologous arms, restriction sites, and a sequence matching the 5' end of the gene. Primer design schematic for the **3,394-bp** target gene cloning. Colony PCR validation of positive clones produces a **614-bp** fragment.

Supplementary Figure legends

Figure 1.

A-T. Validation of the association between differentially expressed protein levels and survival outcomes in CRC using Kaplan-Meier Plotter. U&V. UALCAN database analysis confirming the expression levels of differentially expressed proteins in cancerous versus adjacent normal tissues.

Figure 2.

A&B. Analysis of SSU72 expression levels in CRC tissues using GSE163974 single-cell sequencing data, TCGA database, and multiple GEO datasets. C. Univariate analysis of clinical factors influencing CRC prognosis (based on sample data from our research cohort). D&E. Tumor cell subpopulations were identified from all cells by CNV and marker annotation, and the relationship between SSU72 expression in specific tumor cell subpopulations and pathological factors was subsequently analyzed based on GSE178318. F-H. Identification of tumor cells from epithelial cells via copy number inference using the GSE132465 dataset. I. Analysis of SSU72 expression in tumor cells across different T stages.

Figure 3.

A-F. Cell annotation and analysis of correlations between SSU72 expression levels and autophagy/apoptosis activity using single-cell datasets GSM5688706 and GSM5688709.

Figure 4.

A&B. Validation of SSU72-overexpressing cell lines by RT-qPCR and Western blot. C. Detection of autophagy- and apoptosis-related protein levels following SSU72 overexpression. D&E. Assessment of proliferation and invasion upon SSU72 overexpression using colony formation and Transwell assays. F&G. Validation of SSU72 knockdown (sequence #) cell lines by RT-qPCR and Western blot. H. Detection of autophagy- and apoptosis-related protein levels following SSU72 knockdown. I&J. Assessment of proliferation and invasion upon SSU72 knockdown using colony formation and Transwell assays. K. RT-qPCR detection of XBP1u and XBP1s. L. Western blot analysis of ATF6 p90 (full-length) and p50. M&N. Detection of ER level changes following sh-SSU72# and SSU72 overexpression.

Figure 5.

Construction of PERK phosphorylation site mutants: wild-type PERK (A), *S719A* (B).

Figure 6.

Construction of PERK phosphorylation site mutants: *T982A* (A), *S719A/T982A* (B, double mutant).

Figure 7.

Construction of PERK phosphorylation site mutants: *T982E* (A).
